# Chemical CO_2_ fixation by a heterogenised Zn(ii)-hydrazone complex†

**DOI:** 10.1039/d4ra09026h

**Published:** 2025-02-26

**Authors:** Neda Heydari, Rahman Bikas, Tadeusz Lis

**Affiliations:** a Department of Chemistry, Faculty of Science, Imam Khomeini International University Qazvin 34148-96818 Iran bikas@sci.ikiu.ac.ir bikas_r@yahoo.com; b Faculty of Chemistry, University of Wroclaw Joliot-Curie 14 Wroclaw 50-383 Poland

## Abstract

A new Zn(ii) coordination compound, [Zn(HL)(OAc)_2_] (1), with ONN-donor hydrazone ligand (HL = (*E*)-4-amino-*N*′-(1-(pyridin-2-yl)ethylidene)benzohydrazide) was synthesized and structurally characterized by using spectroscopic techniques and X-ray analysis. These analyses indicated that the Zn(ii) ion in the resulting coordination compound is five coordinated and the compound has a free amine functionality on the phenyl ring. Thus, [Zn(HL)(OAc)_2_] (1) was immobilized on the surface of propionylchloride functionalized silica gel through an amidification process *via* the reaction of the aniline part of compound 1 and the acyl chloride group of the support. The synthesized heterogeneous catalyst, Si-[Zn(HL)(OAc)_2_], was characterized by several analytical methods and the results confirmed the successful support of 1 on the surface of the support. Si-[Zn(HL)(OAc)_2_] was used in a chemical CO_2_ fixation reaction and styrene epoxide was used as a model substrate to investigate the catalytic performance of the supported Zn(ii) catalyst. Si-[Zn(HL)(OAc)_2_] can efficiently catalyze the formation of cyclic carbonate from the reaction of epoxide and CO_2_ in the presence of TBAB as a co-catalyst.

## Abstract 2

Synthesis and characterization of a heterogeneous catalyst based on a silica supported Zn(ii)–hydrazone complex and its activity in the chemical CO_2_ fixation reaction have been reported.

## Graphical abstract

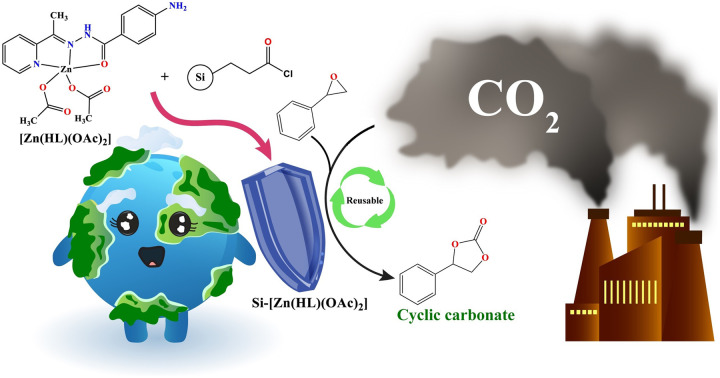

## 1. Introduction

Human activities on the planet have caused carbon dioxide to accumulate significantly in the earth's atmosphere and caused increased concerns about climate change.^1^ These concerns have caused research on the absorption and use of carbon dioxide to reduce greenhouse gases. One of the innovative strategies is the chemical fixation of carbon dioxide and its conversion into other valuable chemical products^2^ such as polymers, fuels, carboxylic acids and cyclic carbonates.^3^ CO_2_ fixation can be done in the presence or absence of catalysts. Transition metal complexes (especially zinc, ruthenium and copper) are important catalysts for chemical CO_2_ fixation, and they play a key role in converting CO_2_ to other compounds under mild conditions.^4^ These catalysts have high ability to activate carbon dioxide and increase the efficiency of the formation of various organic compounds.^5^ Carbon dioxide can be converted to methane or carbon monoxide through electrolysis,^6^ or it can be converted to various carbonates by reacting with hydroxides and oxides of alkali metals.^7^ In CO_2_ fixation in the absence of a catalyst, the chemical processes are not carried out under mild conditions and the use of high pressure and high temperature are required for converting CO_2_ to other materials.^8^ Thus, the use of catalysts and performing CO_2_ fixation reaction under mild condition has attracted much attention during recent decades. The reaction of CO_2_ with epoxides in the presence of a catalyst and converting them to cyclic carbonates is one of the most interesting and efficient methods in chemical CO_2_ fixation^9^ because cyclic carbonates are useful intermediates in the chemical industry, and they have several applications in various fields.^10^

The use of zinc coordination compounds as catalyst in chemical fixation of CO_2_ has attracted high attention due to adjustable reactivity, low cost and abundance of zinc compounds.^11^ Zinc, as a good Lewis acid, can efficiently interact with carbon dioxide and activate it to involve in CO_2_ fixation reactions and this matter considerably facilitates efficient conversion of CO_2_ into other organic compounds.^12^ The mechanism of this process mainly includes the coordination and activation of carbon dioxide to the zinc core of coordination compounds and the formation of active intermediates that can easily react with other suitable reagents and convert to the final products.^13^ The coordination environment of Zn(ii) ion and the structure of organic ligands can considerably influence on the rate of the catalytic reactions and selectivity of the product.^14^ Thus, several studies by using various Zn(ii) coordination compounds should be done to increase our knowledge about the details of such transformations and to design efficient catalysts for CO_2_ fixation under milder and safer conditions. Both homogeneous and heterogeneous catalytic systems have been used for chemical CO_2_ fixation reaction^15^ but, the use of heterogeneous systems has some advantages like easy separation, simple recovery process and reusability of the catalyst^16^ which make them interesting and attractive materials for industrial applications. Therefore, developing heterogeneous catalytic systems based on coordination compounds can be considered as one of the top research fields in catalysis science and technology and also in CO_2_ fixation reactions.^17^

Hydrazone ligands are a type of famous nitrogen and oxygen donor ligands in Schiff base family which are synthesized from the reaction of hydrazides (R–CO–NH–NH_2_) and appropriate aldehyde/ketone derivatives.^18^ Hydrazone ligands are known for their versatile coordination chemistry and tunable properties, and they mostly form stable coordination compounds.^19^ Their coordination compounds have several applications in biological, chemical and material sciences and usually show high catalytic activity and stability in various reactions.^20^ Although hydrazone coordination compounds are known as homogeneous catalysts, during recent years several strategies have been used for converting them into heterogeneous systems.^21^ Among them, introducing a suitable functional group on the structure of the hydrazone ligands and connecting them onto the surface of a heterogeneous substrate (like silica) is the most successful way.

In this report, we describe the synthesis, crystal structure, spectroscopic properties and catalytic activity of a new Zn(ii) coordination compound with a hydrazone ligand. Since there is a free aniline functionality on the structure of the synthesized compound, it was converted to a heterogeneous catalyst by supporting on the surface of functionalized silica gel. The resulting heterogeneous compound was characterized by various analytical methods and its catalytic activity was investigated in chemical fixation of CO_2_.

## 2. Experimental

### 2.1. Materials

2-Acetylpyridine, 4-aminobenzoic hydrazide, propionylchloride-functionalized silica gel (≈1 mmol g^−1^ loading), zinc(ii) acetate dihydrate and other chemicals were purchased from Sigma-Aldrich. Solvents with the highest grade were prepared by Merck and used as received. The ligand (HL) was prepared according to our previous report.^22^

### 2.2. Characterization methods

Powder X-ray diffraction analyses were performed on a Bruker D8 Advance PXRD instrument. Thermal gravimetric analysis was recorded on a TA Q600 instrument (at 25–1000 °C). Scanning electron microscopy and energy-dispersive spectroscopy were taken with a TESCAN MIRA III instrument. Elemental analyses (carbon, hydrogen and nitrogen) were provided using a Carlo ERBA Model EA1108 analyzer and the zinc content of the compounds was measured by a Varian AA-220 atomic absorption instrument. The UV-vis spectra were obtained by using a thermos-spectronic Helios Alpha spectrophotometer (200–800 nm). Photoluminescence (PL) spectra were recorded on a Hitachi F2700 spectrophotometer. Diffuse-reflectance UV-vis spectroscopy was measured with a Sinco S4100 instrument (200–1000 nm). NMR spectra were recorded on a Bruker DRX-300 spectrometer (at 250 MHz). FT-IR spectra were carried out as KBr disks with a Bruker Tensor27 spectrophotometer (4000–400 cm^−1^). An Agilent 7890B gas chromatography (GC) instrument equipped with FID detector (temperature 300 °C) and a HP-5 capillary column (phenyl methyl siloxane 30 m × 320 μm × 0.25 μm) was used to check the progress of the catalytic reactions.

### 2.3. Synthesis of [Zn(HL)(OAc)_2_] (1)

**Scheme 1:**
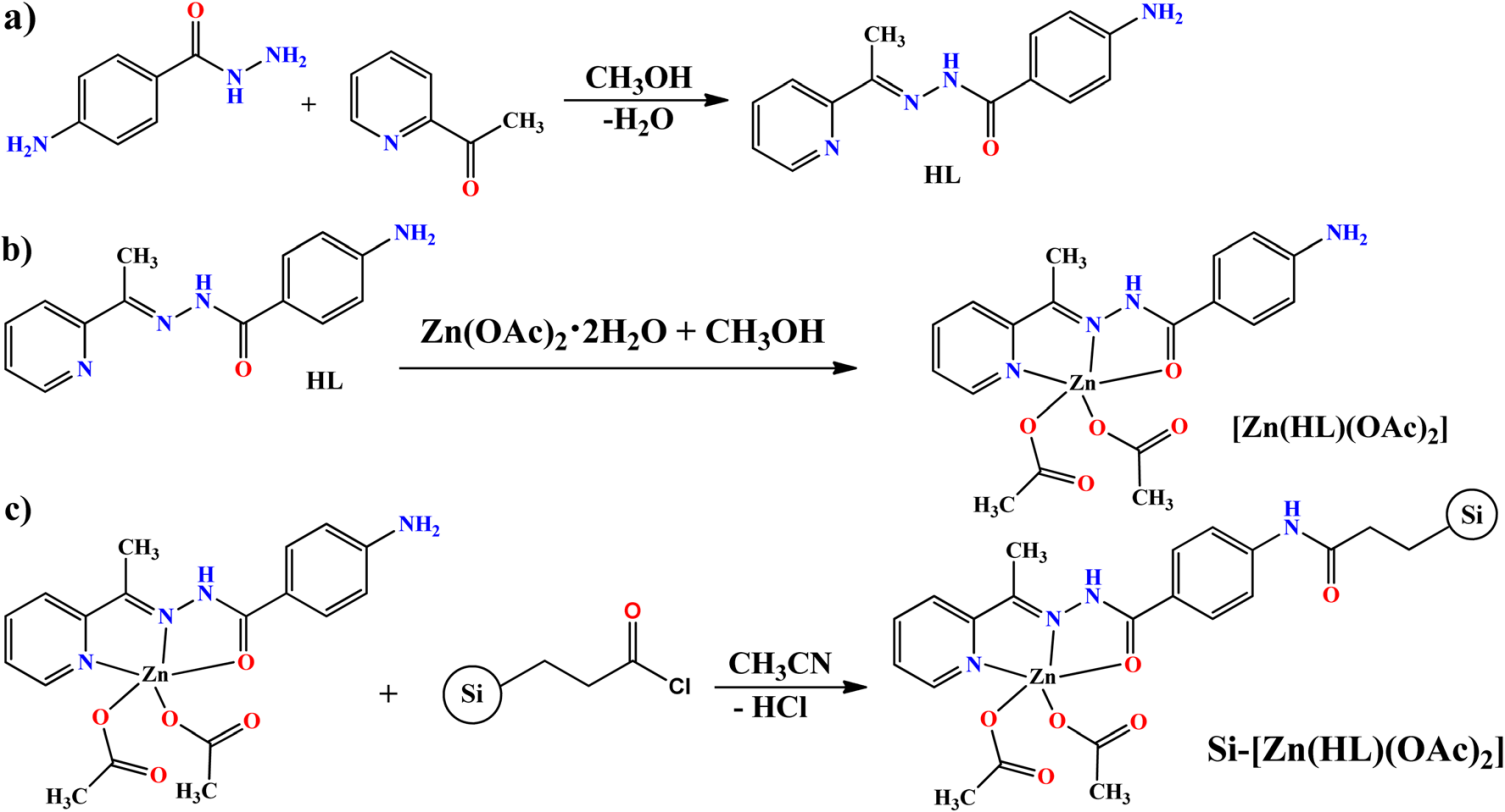
Synthesis pathway of (a) HL; (b) [Zn(HL)(OAc)_2_] (1); and (c) heterogenised Zn catalyst (Si-[Zn(HL)(OAc)_2_]).

For the synthesis of zinc complex, Zn(OAc)_2_·2H_2_O (0.44 g, 2.0 mmol) was added to the solution of HL (0.508 g, 2.0 mmol) in 25 ml methanol (see Scheme 1). After a short time, the color of the solution turned to yellow and the mixture was stirred for 2 hours at RT. Then, the resulting mixture was heated and stirred under reflux condition for 4 hours. The volume of the solvent was reduced by vacuum and the resulting yellow precipitate was isolated by filtration and washed with methanol. The obtained powder was dissolved in hot methanol and the air stable yellow block crystals, suitable for X-ray analysis, were obtained by evaporating of the solvent. Yield: 79.4% (0.695 g). Anal. calc. For C_18_H_20_N_4_O_5_Zn (437.75 g mol^−1^): C, 49.39; H, 4.61; N, 12.80; Zn, 14.94%. Found: C, 49.45; H, 4.65; N, 12.74; Zn, 14.80%. FT-IR (KBr, cm^−1^): 3555 (m, br), 3432 (s, br), 3333 (s, br), 3205 (s, br), 2996 (w), 2970 (w), 2926 (w), 1635 (s), 1617 (s), 1560 (s), 1513 (s), 1473 (s), 1397 (*vs.*), 1335 (s), 1287 (*vs.*), 1182 (*vs.*), 1166 (*vs.*), 1123 (s), 1017 (s), 975 (w), 935 (w), 902 (w), 845 (*vs.*), 785 (s), 767 (s), 745 (w), 672 (s), 637 (m), 626 (s), 567 (s), 551 (m), 507 (s), 477 (w), 430 (w), 417 (w). ^1^H NMR (250 MHz, DMSO-d_6_, TMS, ppm): *δ* = 1.83 (s, 6H); 2.66 (s, 3H), 5.53 (s, 2H); 6.52 (m, 2H), 7.27, (s, 1H), 7.51 (s, 1H), 7.85 (m, 2H), 7.04 (m, 1H) and 8.45 ppm (s, 1H). ^13^C NMR (62.9 MHz, DMSO-*d*_6_, 25 °C, ppm): *δ* = 12.4, 22.3, 113.0, 122.0, 123.8, 125.0, 130.1, 140.6, 146.9, 148.6, 150.8, 151.8, 160.4, 174.0 and 174.9 ppm. UV-vis (2.5 × 10^−5^ M, CH_3_OH): *λ*_max_ (*ε*, M^−1^ cm^−1^): 222 (27 600), 285 (27 300), 360 nm (60 500).

### 2.4. Synthesis of heterogeneous catalyst (Si-[Zn(HL)(OAc)_2_])

Functionalized silica gel with propionylchloride was used to prepare silica supported Zn catalyst *via* amidification reaction (see Scheme 1). For this matter, 0.175 g of 1 (0.40 mmol) and 0.40 g of silica gel (≈1 mmol g^−1^ loading) were reacted in 10 ml of dried acetonitrile. The reaction mixture was stirred and heated at 80 °C for one day. The resulting yellow solid was filtered, washed several times with distilled water and dried at air. Yield: 94.58% (0.530 g). FT-IR (KBr, cm^−1^): 3546 (m, br), 3447 (m, br), 3416 (m, br), 3232 (m, br), 1725 (m), 1637 (s), 1616 (s), 1601 (s), 1561 (w), 1517 (w), 1494 (m), 1461 (m), 1424 (w), 1400 (w), 1359 (s), 1317 (m), 1288 (m), 1062 (br), 945 (m), 909 (m), 805 (s), 771 (s), 745 (w), 708 (w), 684 (w), 631 (m), 468 (w, br).

### 2.5. Catalytic studies

For investigating the catalytic activity of Si-[Zn(HL)(OAc)_2_] in chemical CO_2_ fixation, the reactions were done in two necked round bottom flasks connected to a condenser. The flask was charged with styrene epoxide (5.0 mmol; as substrate), solvent (10.0 ml; CH_3_CN, EtOH, THF or CHCl_3_), and tetrabutylammonium bromide as a co-catalyst (0.1–2.0 mmol). As a catalyst, Si-[Zn(HL)(OAc)_2_] (15.0 mg, 9.20 μmol Zn(ii) ion) was added to the reaction mixture and CO_2_ was bubbled from the solution at atmospheric pressure to have a CO_2_ saturated solution. Then, a CO_2_ balloon was connected to the top of the condenser. The flask was inserted into an oil bath at the desired temperature and the mixture was continuously stirred. The progress of the reactions was monitored by TLC technique and GC instrument. The carbonate product was isolated from the reaction mixture by solvent extraction method and after purification processes, it was characterized by spectroscopic methods. The heterogeneous catalyst was simply recovered by filtration (or decantation) and reused in the next rounds.

### 2.6. X-ray crystallographic study and Hirshfeld surface analysis

Single crystal of [Zn(HL)(OAc)_2_] was selected for X-ray analysis and its data were collected at 100 K using a Kuma KM-4 diffractometer with monochromatic Mo Kα radiation (*λ* = 0.71073 Å). Unit cell refinement, data collection, data reduction and absorption correction were performed with the CrysAlisPro software package.^23^ The data were obtained by ω-scan mode. The structure of the compound was refined by full matrix least squares using SHELX-2013 software.^24^ The crystallographic data and important refinement parameters are collected in Table 1. Crystal-Explorer17 program^25^ was used to investigate the intermolecular interactions in the structure of [Zn(HL)(OAc)_2_].

**Table 1:** Crystal data and structure refinement parameters for [Zn(HL)(OAc)_2_]

Formula	C_18_H_20_N_4_O_5_Zn
MW/g mol^−1^	437.75
Crystal shape, color	Block, yellow
Crystal size/mm	0.36 × 0.27 × 0.15
*T*/K	100
Crystal system	Triclinic
Space group	*P*1̄
*a*/Å	9.037(2)
*b*/Å	10.049(3)
*c*/Å	10.795(3)
*α*/°	80.44(4)
*β*/°	78.53(4)
*γ*/°	79.74(3)
*V*/Å^−3^	936.7(5)
*Z*	2
*D*_calc_/g cm^−3^	1.552
μ/mm^−1^	1.35
*T*_min_, *T*_max_	0.672, 0.853
*F*(000)	452
*θ* range/°	2.8–25
*R*_int_	0.042
*R*[*F*^2^ > 2*σ*(*F*^2^)]	0.051
*wR*(*F*^2^)	0.142
*S*	1.04
Abs. correction	Analytical
Hydrogen refinement	Mixed
Measured reflections	7344
Independent reflections	3855
Reflections with *I* > 2*σ*(*I*)	3579
Parameters	268
Restraints	0
Δ*ρ*_max_/Δ*ρ*_min_/e Å^−3^	0.50/−0.85

## 3. Results and discussion

### 3.1. Synthesis of HL, [Zn(HL)(OAc)_2_] (1) and supported catalyst (Si-[Zn(HL)(OAc)_2_])

[Zn(HL)(OAc)_2_] was obtained as yellow block single crystals by slow evaporating of the methanolic solution. The structure of the resulting crystals was determined by X-ray analysis and the product was also characterized by NMR, FT-IR, PL, UV-vis, TGA and elemental analyses. By having a free NH_2_ group in the structure of this compound, [Zn(HL)(OAc)_2_] (1), we supported it on the surface of silica gel. The reaction between the amine group of [Zn(HL)(OAc)_2_] and propionylchloride group of the functionalized silica gave the supported catalyst (Si-[Zn(HL)(OAc)_2_]) as a yellowish powder which was characterized by atomic absorption, FT-IR, UV-DRS, TGA, SEM, EDX and XRD techniques.

### 3.2. X-ray structure of [Zn(HL)(OAc)_2_] (1) and Hirshfeld surface analysis

**Fig. 1:**
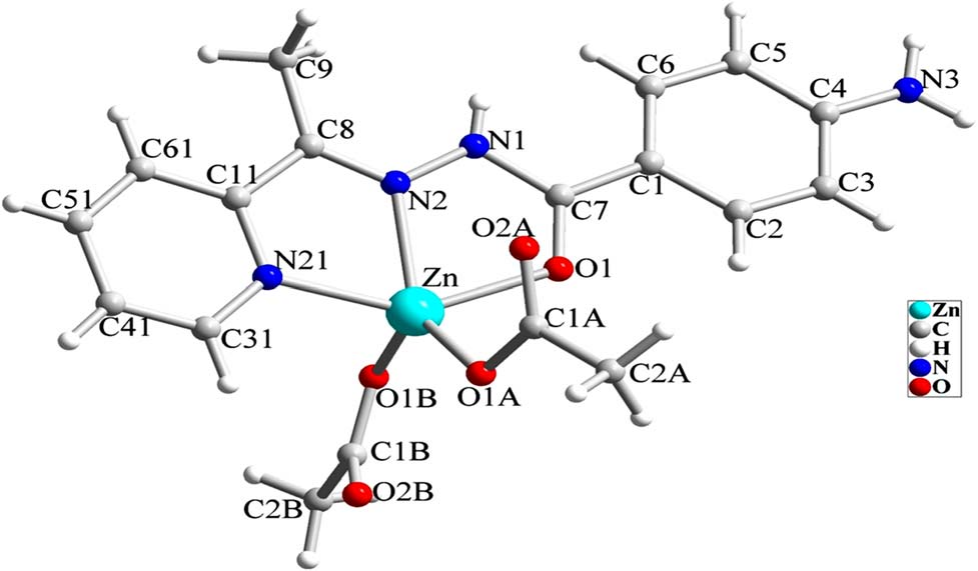
Molecular structure of [Zn(HL)(OAc)_2_] (1).

**Fig. 2:**
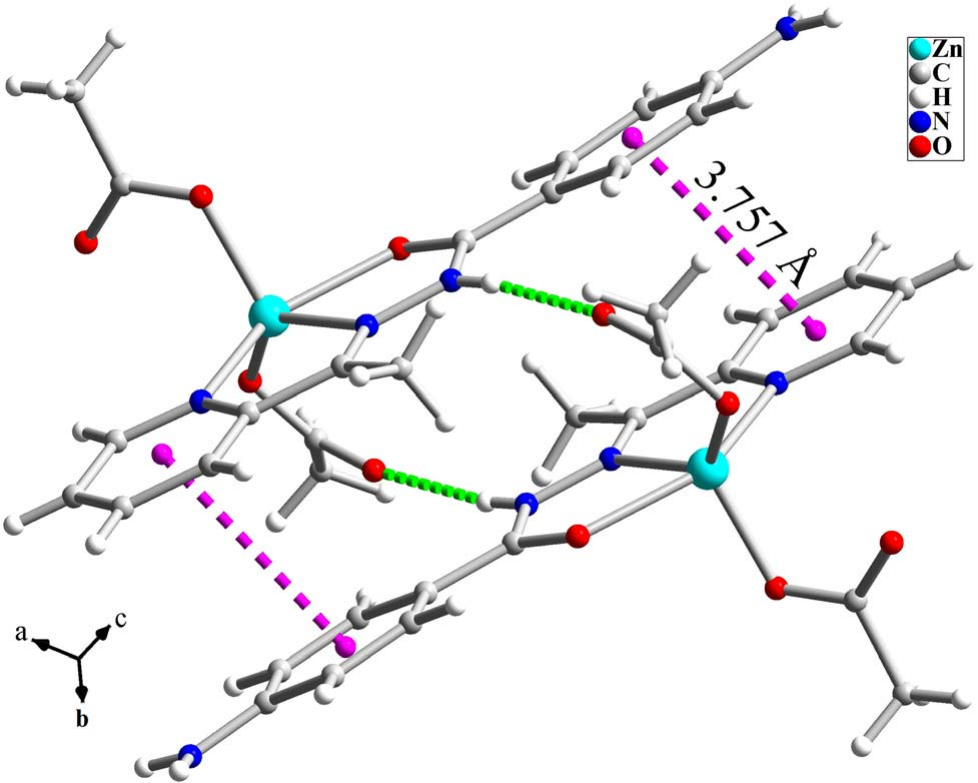
Hydrogen bond interactions (N–H⋯O = green dashed line) and π⋯π stacking (pink dashed line) in the crystal of [Zn(HL)(OAc)_2_] (1).

**Fig. 3:**
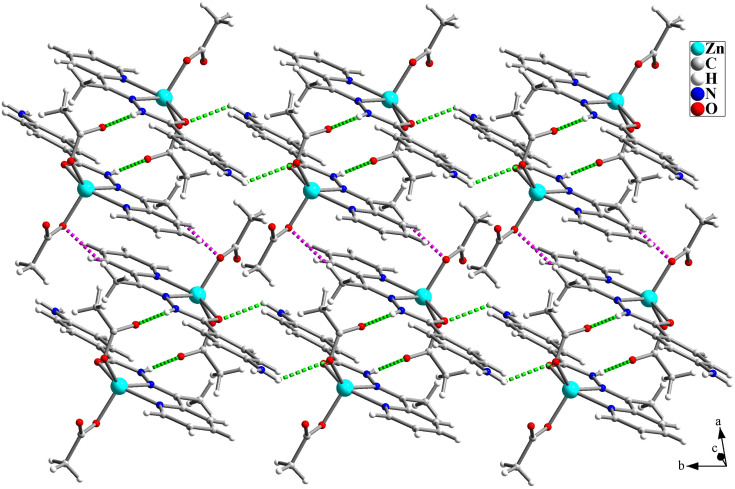
Hydrogen bond interactions (N–H⋯O = green and C–H⋯O = pink) in the structure of [Zn(HL)(OAc)_2_] (1).

The structure of [Zn(HL)(OAc)_2_] is shown in Fig. 1 and Table 2 shows the selected bond lengths and angles around the Zn(ii) in this structure. According to X-ray diffraction studies, the product is a neutral mononuclear Zn(ii) coordination compound with a general formula of [Zn(HL)(OAc)_2_] and crystallizes in triclinic system with *P*1̄ space group. The Zn(ii) atom is five-coordinated and pursues a distorted square pyramidal [ZnN_2_O_3_] geometry that was inferred by calculating the values of Addison parameter (*τ* = 0.179). In the structure of this compound, the ligand acts as a NNO-donor ligand and is coordinated to the Zn(ii) center as a neutral ligand in amidic form (HL). The imine and pyridine nitrogen atoms (N2 and N21) together with the amidic oxygen atom (O1) of HL are coordinated to the Zn(ii) center and fill three corners of the square plane. The remaining corner is filled by the oxygen atom of the acetate ligand (O1A). In the equatorial plane, the distances of Zn–N2, Zn–N21, Zn–O1 and Zn–O1A are 2.118(2), 2.142(3), 2.177(2) and 1.954(2) Å, respectively. These bond lengths are in the normal range observed in the similar Zn(ii) complexes.^26^ The axial position is filled by the oxygen atom of the second acetate ligand (O1B) with the Zn–O1B distance of 1.963(2) Å. In the crystal of [Zn(HL)(OAc)_2_], there are several intermolecular hydrogen bonds and π⋯π stacking interactions (see Fig. 2 and 3) which stabilize the crystal. Hydrogen bonding data are given in Table 3.

**Table 2:** Selected bond lengths and angles in the crystal structure of [Zn(HL)(OAc)_2_]

Bond	Length/Å	Bond	Angle/°
Zn–O1A	1.954(2)	O1A–Zn–O1B	116.97(9)
Zn–O1B	1.963(2)	O1A–Zn–N2	136.36(9)
Zn–N2	2.118(3)	O1B–Zn–N2	105.63(9)
Zn–N21	2.142(3)	O1A–Zn–N21	106.11(9)
Zn–O1	2.177(2)	O1B–Zn–N21	99.12(9)
C7–O1	1.244(4)	N2–Zn–N21	74.51(10)
C7–N1	1.380(4)	O1A–Zn–O1	93.43(9)
N1–N2	1.365(4)	O1B–Zn–O1	94.93(9)
N2–C8	1.290(4)	N2–Zn–O1	73.15(9)
C4–N3	1.355(4)	N21–Zn–O1	147.15(9)

**Table 3:** Hydrogen bonding interactions (Å, °) in the crystal of [Zn(HL)(OAc)_2_]

D–H···A	D–H	H⋯A	D⋯A	D–H···A
N3–H3A⋯O2B^a^	0.85(4)	2.18(4)	3.001(4)	164(3)
N3–H3B⋯O2B^b^	0.87(5)	2.39(4)	3.195(4)	153(4)
N1–H1⋯O2A^c^	0.71(4)	2.08(4)	2.784(4)	171(4)
C41–H41⋯O1A^d^	0.95	2.44	3.380(4)	171

a ^a^Symmetry codes.*x* − 1, *y*, *z* + 1.

b −*x*, −*y*, −*z* + 1.

c −*x*, −*y* + 1, −*z* + 1.

d −*x* + 1, −*y* + 1, −z.

**Fig. 4:**
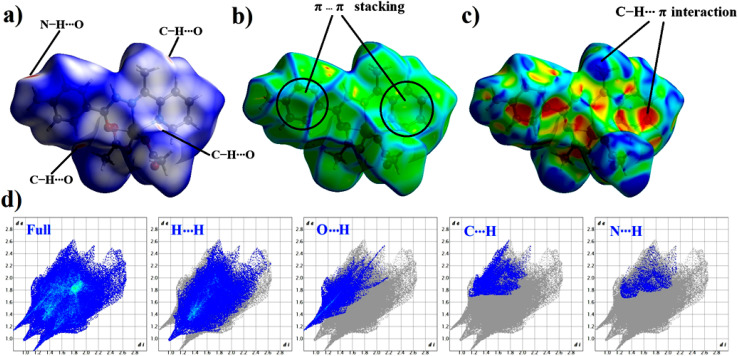
Hirshfeld surface of [Zn(HL)(OAc)_2_] mapped with (a) *d*_norm_, (b) curvedness and (c) shape index (d) fingerprint plots.

**Fig. 5:**
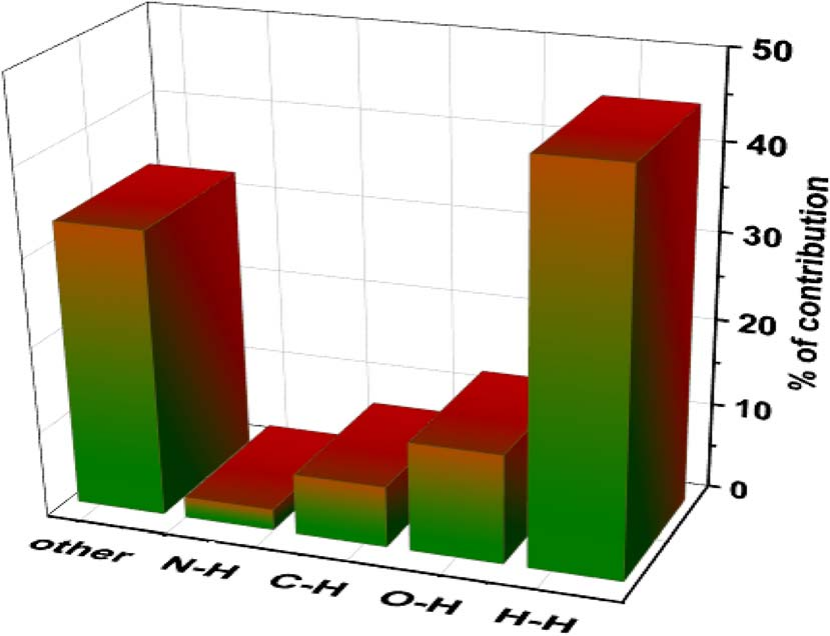
Contribution of intermolecular contacts for Hirshfeld surface of [Zn(HL)(OAc)_2_].

Due to the presence of several intermolecular interactions in the crystal of [Zn(HL)(OAc)_2_], Hirshfeld surface (HS) analysis was used for investigating these interactions in its structure. The Hirshfeld surfaces of [Zn(HL)(OAc)_2_] mapped with (a) *d*_norm_, (b) curvedness and (c) shape index are shown in Fig. 4. The *d*_norm_ Hirshfeld surface is shown in Fig. 4a and indicates the interaction between the closer atoms outside the surface (*d*_e_) and inside the surface (*d*_i_) such as red (close proximity), white (medium proximity) and blue colors (little proximity of the outside atoms).^27^ As shown in Fig. 4a, the *d*_norm_ Hirshfeld surface of [Zn(HL)(OAc)_2_] shows four main red spots which are related to the close proximity such as N–H⋯O and C–H⋯O contacts. The curvedness Hirshfeld of [Zn(HL)(OAc)_2_] is shown in Fig. 4b which includes two flat areas on the curvedness surface showing the presence of π⋯π stacking interactions. The shape index Hirshfeld of [Zn(HL)(OAc)_2_] is shown in Fig. 4c. The presence of blue (bulging) and orange (hollow) regions indicates the presence of intermolecular C–H⋯π interactions. For this crystal, the fingerprint plots and the contribution of some main contacts are shown in Fig. 4d and 5, respectively. The fingerprint plots of [Zn(HL)(OAc)_2_] indicate that H⋯H contacts have the most contribution (45.1%) and the contribution of O⋯H, C⋯H and N⋯H contacts are 12.6%, 7.9% and 2.4%, respectively.

### 3.3. Spectroscopic characterization of the synthesized compounds

#### 3.3.1. FT-IR spectroscopy

The FT-IR spectra of HL, [Zn(HL)(OAc)_2_], starting propionylchloride-functionalized silica gel and heterogeneous catalyst (Si-[Zn(HL)(OAc)_2_]) are shown in Fig. S1–S4,† respectively. In the FT-IR spectrum of HL, the band at 1604 cm^−1^ is attributed to the C=N stretching frequency that is shifted to 1617 cm^−1^ in [Zn(HL)(OAc)_2_] indicating the coordination of HL to Zn(ii) ion through imine nitrogen atom.^28^ The amidic C=O band of HL is appeared as a strong band at 1655 cm^−1^, and it is shifted to 1635 cm^−1^ in the spectrum of [Zn(HL)(OAc)_2_]. It should be noted that the C=O band of acetate groups is overlapped with the amidic C=O, which confirms the coordination of C=O oxygen to the Zn(ii) ion. The NH stretching band in the spectra of HL and [Zn(HL)(OAc)_2_] is observed at 3229 and 3205 cm^−1^, respectively.^29^ The bands related to the NH_2_ group in the spectrum of [Zn(HL)(OAc)_2_] are observed at 3333 and 3432 cm^−1^ and these bands are located at 3367 and 3338 cm^−1^ in the spectrum of HL. In the FT-IR spectrum of Si-[Zn(HL)(OAc)_2_], there are three strong and broad bands at 1062, 805 and 468 cm^−1^ which are related to the vibration of the silica support.^30^ The broad band at 3416 cm^−1^ is due to the OH group in the structure of supported catalyst. Two absorption bands at 1637 and 1601 cm^−1^ are respectively assigned to the amide C=O and imine C=N groups of the supported [Zn(HL)(OAc)_2_]. The FT-IR spectra of these compounds confirm the successful support of [Zn(HL)(OAc)_2_] on silica support.

#### 3.3.2. NMR spectroscopy

The ^1^H NMR spectrum of [Zn(HL)(OAc)_2_] in DMSO-*d*_6_ solvent is shown in Fig. S5.† In this spectrum, the peaks at *δ* = 1.83, 2.66 and 5.53 ppm are related to the –CH_3_ of acetate anions, –CH_3_ of the acetyl group, and –NH_2_ of the aniline ring, respectively. The peaks related to aromatic hydrogens are observed as six peaks in the range of 6.5–8.5 ppm. There are fifteen unique peaks in the ^13^C NMR spectrum of [Zn(HL)(OAc)_2_] (Fig. S6†) which confirms its structure and the high purity of the product. In this spectrum, the peaks at *δ* = 12.4, 22.3, 146.9, 148.6 and 160.4 ppm are attributed to the –CH_3_ (acetyl group), –CH_3_ (acetate anions), imine C=N, C–NH_2_ and amide C=O groups, respectively. The peaks observed at *δ* = 174.0 and 174.9 ppm are related to the carboxyl group of the acetate ligands^31^ which indicate two acetate groups have slightly different conditions and one of the carbon peaks is observed at a slightly different position.

#### 3.3.3. UV-vis and photoluminescence spectra

**Fig. 6:**
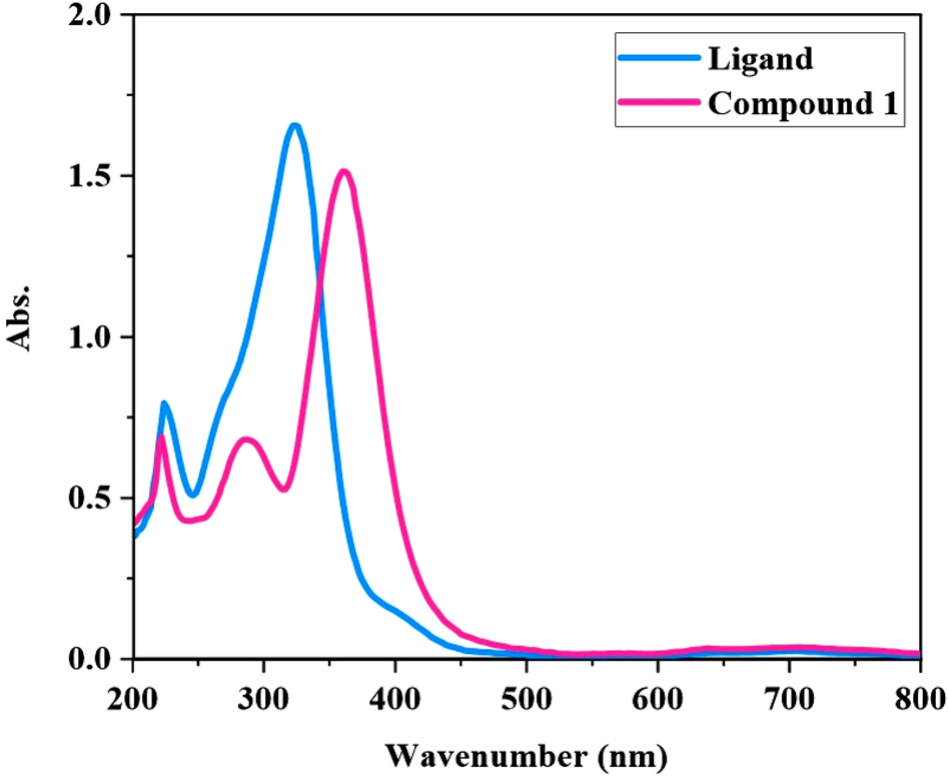
UV-vis spectra of HL and [Zn(HL)(OAc)_2_] (2.5 × 10^−5^ M) in methanol.

Fig. 6 shows the UV-vis spectrum of [Zn(HL)(OAc)_2_] and HL recorded in MeOH solution. In the spectrum of HL, the band at *λ*_max_ = 225 nm is due to π → π* transition and the band at 320 nm is related to a mixture of π → π* and n → π* transitions. In the spectrum of [Zn(HL)(OAc)_2_], the band at *λ*_max_ = 222 nm and also the band at 285 nm are related to intraligand transitions. The broad band of the free ligand, observed at about 320 nm, has been eliminated in the spectrum of [Zn(HL)(OAc)_2_], which indicates the coordination of non-bonding electrons of C=N and C=O groups to the metal ion and due to this, the intraligand n → π* transitions are eliminated in the spectrum of complex. In the spectrum of [Zn(HL)(OAc)_2_], a new strong and relatively broad band is observed in the range of 330–450 nm with *λ*_max_ = 360 nm, which is devoted to the charge transfer transitions. The slight shift of π → π* transitions, the elimination of intraligand n → π* transitions and the observation of charge transfer transitions in the spectrum of [Zn(HL)(OAc)_2_] clearly confirm the coordination of HL to the Zn(ii) ion.

**Fig. 7:**
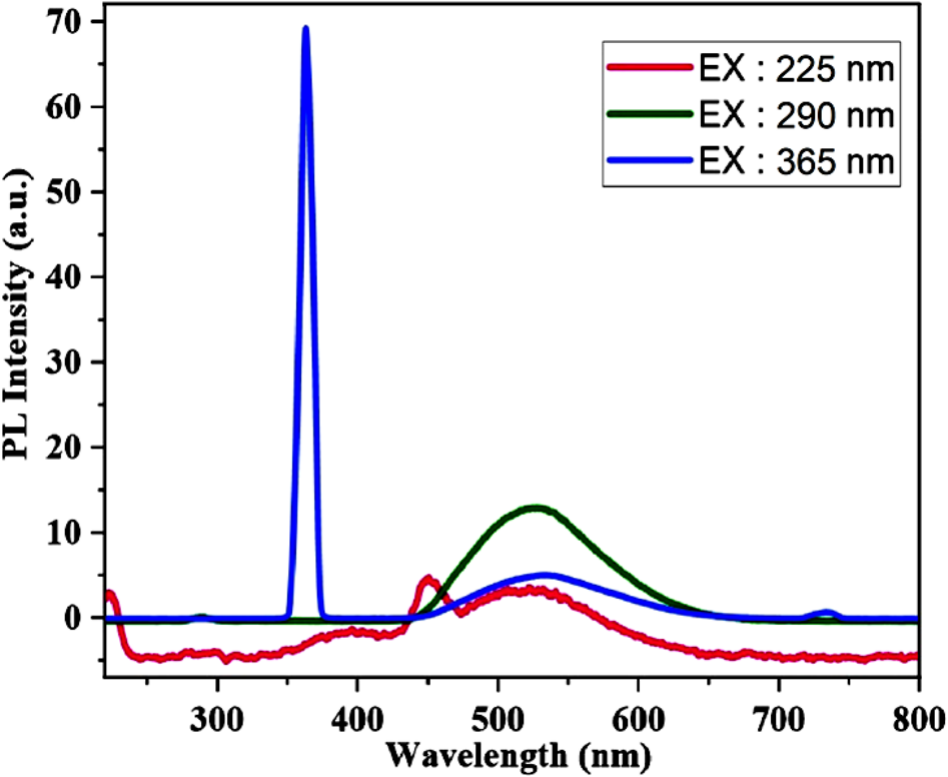
The PL spectra of [Zn(HL)(OAc)_2_] at the different excitation wavelengths.

The emission spectra of [Zn(HL)(OAc)_2_] in the range of 220–800 nm were recorded at the excitation wavelengths (225, 290 and 365 nm), which are shown in Fig. 7. The spectra display emission bands and in all the excitation wavelengths, broad emission bands appear in the range of 450–600 nm with maximum intensity at 522, 525 and 530 nm. There are two weak emissions at about 450 and 730 nm by excitations at 225 and 365 nm, respectively, which are the second order Rayleigh scattering of these excitations.^32^ Zn(ii) compounds do not exhibit d–d transitions because of their d^10^ configuration. As a result, the observed peaks are the result of electron relaxation from higher to lower energy levels.

The UV-DRS spectrum of Si-[Zn(HL)(OAc)_2_] was recorded in the range of 300–1000 nm (see Fig. S7†). There are two obvious absorption bands at 361 and 411 nm in this spectrum which are in agreement with the UV-vis spectrum of [Zn(HL)(OAc)_2_] in methanol solution. These bands are related to intraligand and charge transfer transitions, respectively. The slight differences between solid state UV-DRS and solution UV-vis spectra are related to the phase and environmental differences and also to the changes in the intermolecular interactions in these two different conditions.

#### 3.3.4. TGA analysis

Thermal stability of [Zn(HL)(OAc)_2_] and Si-[Zn(HL)(OAc)_2_] was investigated by TGA analysis and the obtained curves are shown in Fig. S8 and S9,† respectively. As can be seen in Fig. S8,† [Zn(HL)(OAc)_2_] decomposes in four steps and the first step in the range of 100–200 °C is related to the elimination of acetate ligands. The organic ligand deposes in two steps in the range of 250–400 °C. The decomposition process continues up to 1000 °C and the total weight loss is equal to 81.67% of the starting mass. It is predictable that ZnO is the final product of the thermal decomposition process. TGA curve of Si-[Zn(HL)(OAc)_2_] (Fig. S9†) shows the elimination of weight in three steps which their range are close to the steps observed in TGA curve of [Zn(HL)(OAc)_2_]. This matter confirms that [Zn(HL)(OAc)_2_] has successfully supported on the surface of the silica gel. The total mass elimination in Si-[Zn(HL)(OAc)_2_] is about 30.77% of the starting mass and the remaining mass is mainly related to the formation of a mixture of SiO_2_ and ZnO.

#### 3.3.5. EDX analysis

**Fig. 8:**
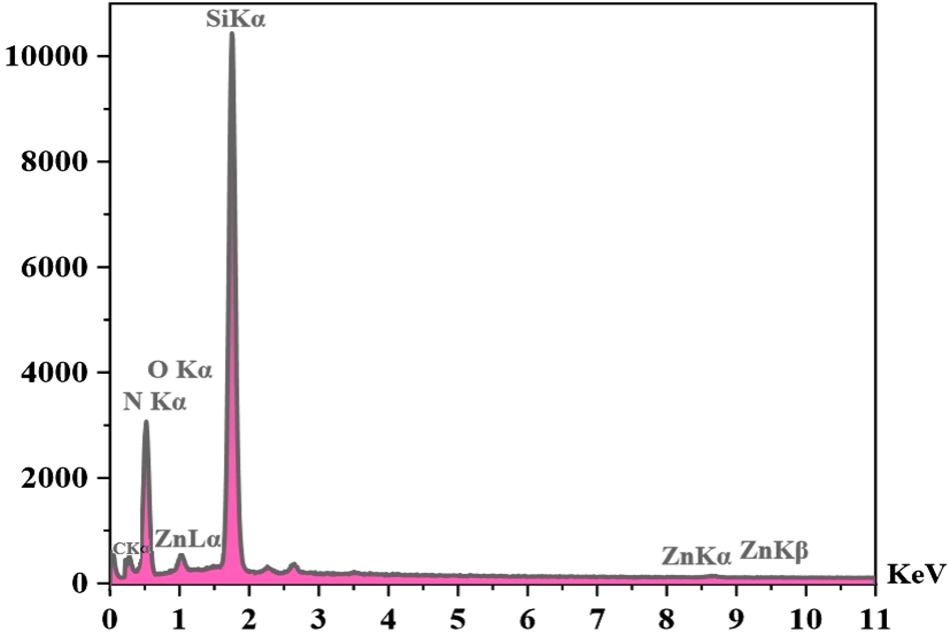
EDX spectrum of Si-[Zn(HL)(OAc)_2_].

**Fig. 9:**
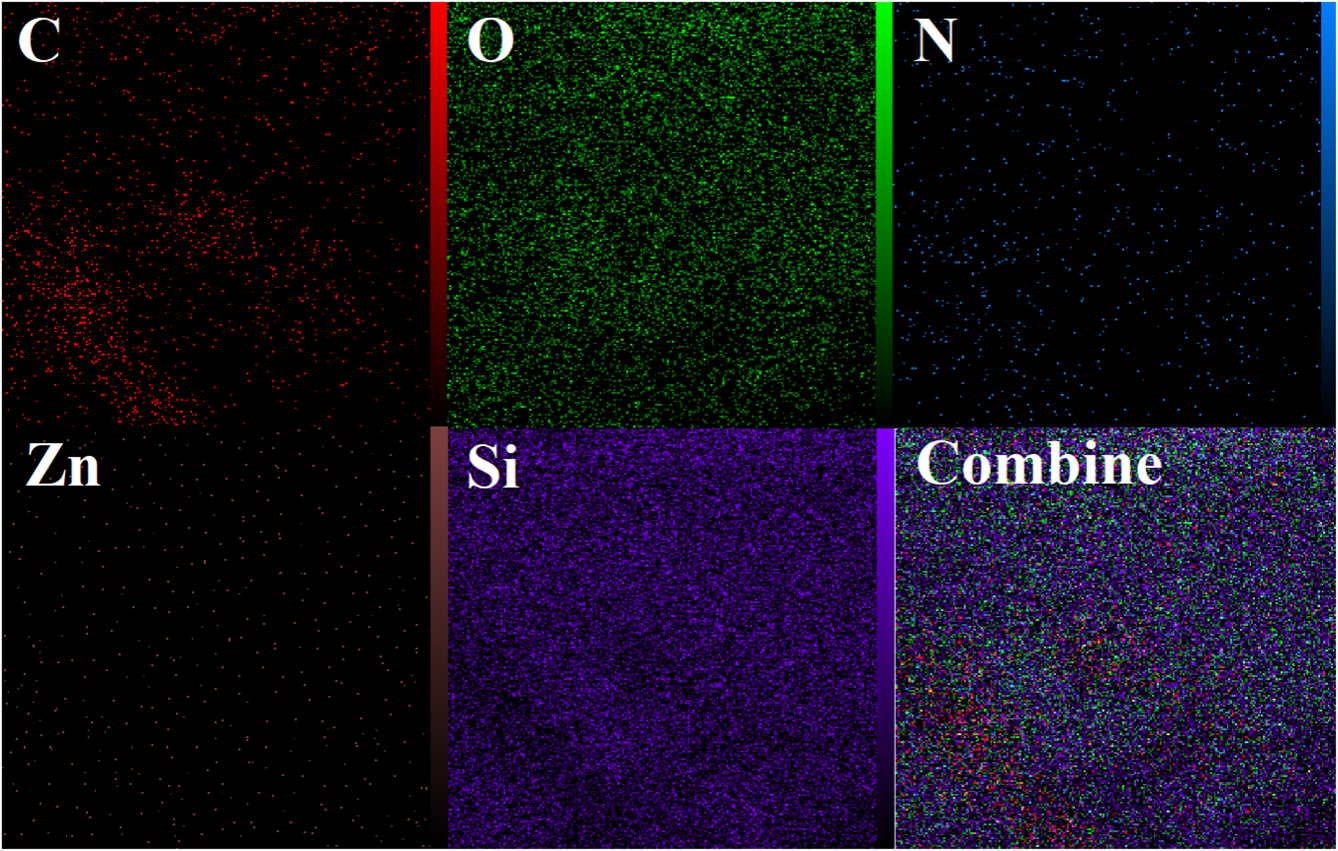
EDX mapping images of Si-[Zn(HL)(OAc)_2_].

The elemental structure of Si-[Zn(HL)(OAc)_2_] was revealed by EDX analysis (see Fig. 8) and the results confirm the presence of the predictable elements (zinc, carbon, nitrogen, oxygen and silicon) in the composition of the surface of this compound. The EDX mapping images indicate that these species (Zn, C, N, O and Si) have been uniformly distributed in the texture of the heterogenised catalyst (see Fig. 9).

#### 3.3.6. SEM analysis

**Fig. 10:**
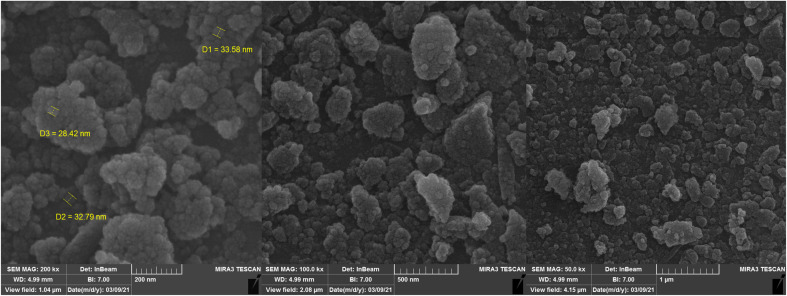
SEM images of the heterogeneous catalyst (Si-[Zn(HL)(OAc)_2_]).

The particle size and the morphology of Si-[Zn(HL)(OAc)_2_] were determined by SEM images. According to the SEM images (see Fig. 10), the particles have spherical shape and their size on the surface is in the nanometer range (approximately 28–50 nm).

#### 3.3.7. XRD analysis

**Fig. 11:**
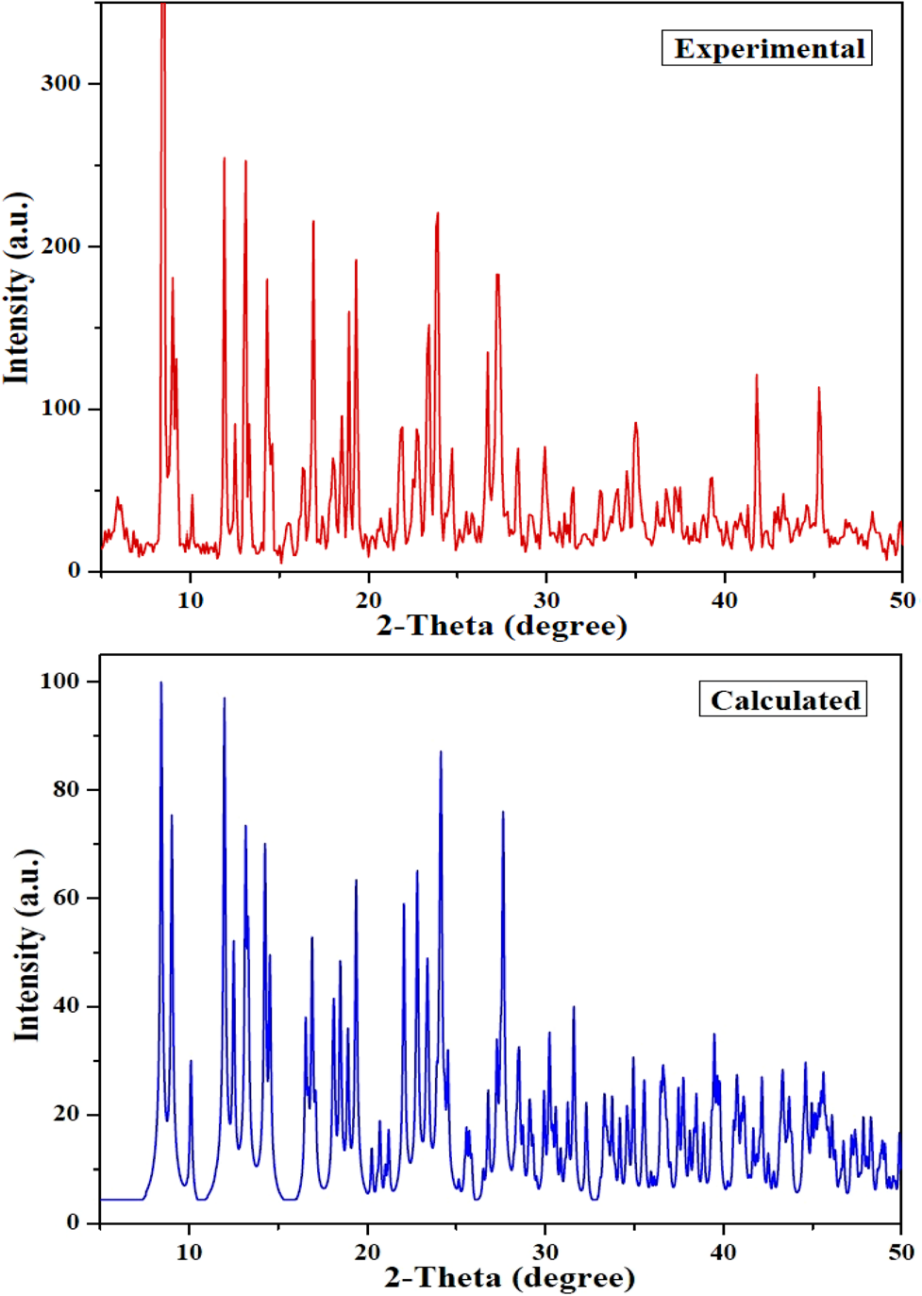
Experimental (top, red) and calculated (bottom, blue) XRD pattern of [Zn(HL)(OAc)_2_].

XRD pattern of bulk crystals of [Zn(HL)(OAc)_2_] was recorded and the resulting pattern is shown in Fig. 11. The resulting XRD pattern has high similarity with the calculated pattern (obtained from single crystal X-ray analysis) which indicates the structure of bulk crystals is the same as the investigated crystal by SC-X-ray analysis. The XRD patterns of the starting silica support and Si-[Zn(HL)(OAc)_2_] are shown in Fig. S10 and S11,† respectively. The broad peak at 2*θ* = 22.6° and the crystalline peaks at 2*θ* = 28.8, 42.8, 49.2, 67.3 and 74.5° are devoted to the silica gel and are in accordance with its XRD pattern.^33^ The new and high crystalline peaks observed in the XRD pattern of Si-[Zn(HL)(OAc)_2_] at 2*θ* = 10.2, 12.2, 16.1, 18.3, 26.7, 29.9, 32.7, 33.9, 38.7, 44.9, 47.9, 48.2, 52.1, 52.8, 56.4 and 59.1° are related to [Zn(HL)(OAc)_2_] and confirm the successful supporting of [Zn(HL)(OAc)_2_] on the surface of silica.

### 3.4. Catalytic CO_2_ fixation

**Scheme 2:**
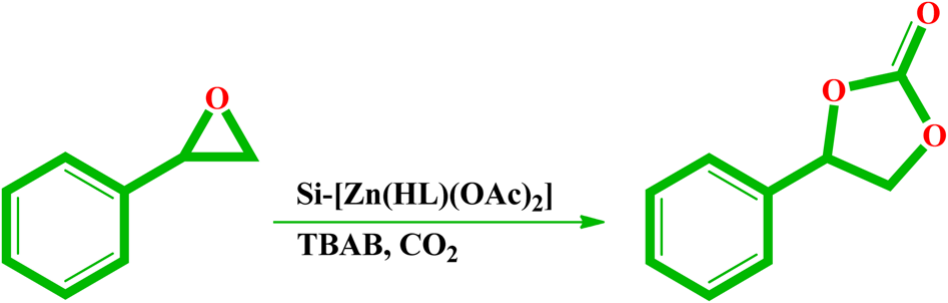
The reaction pathway of CO_2_ coupling by styrene epoxide in the presence of Si-[Zn(HL)(OAc)_2_].

The results of various analyses confirmed the successful supporting of [Zn(HL)(OAc)_2_] on the silica support. The loading of Zn(ii) ion by atomic absorption analysis was obtained as 4.01 wt% which is equal to 0.613 mmol g^−1^ of the catalyst. Due to the high activity of Zn(ii) compounds in the chemical CO_2_ fixation reactions, the catalytic activity of Si-[Zn(HL)(OAc)_2_] was investigated in the formation of cyclic carbonates from the reaction of CO_2_ and epoxide. The catalytic reactions were done in a round bottom flask containing Si-[Zn(HL)(OAc)_2_] (as catalyst), styrene epoxide (as substrate), solvent (CH_3_CN, EtOH, THF or CHCl_3_), and tetrabutylammonium bromide (TBAB, as co-catalyst) under CO_2_ rich atmosphere (see Scheme 2). The control experiments in the absence of the catalyst or co-catalyst indicated there are low amounts of the cyclic product in the absence of one of them. When both the catalyst and co-catalyst were simultaneously employed, the rate of the reaction was considerably increased, and styrene epoxide was converted to phenylethylene carbonate. Thus, the effect of the ratio of co-catalyst was studied by using various amounts of TBAB in the presence of constant amounts of the other reagents. The results of these studies are collected in Table 4 and indicate 1.0 mmol of TBAB has better performance than others. Temperature of the reaction was also investigated, and the best result was obtained at 70 °C in CH_3_CN solvent. Although the catalyst in this reaction is heterogeneous, we interested to investigate the effect of the solvent on its performance by considering the fact that solvent can considerably increase the interaction between substrate, co-catalyst and catalyst. Moreover, CO_2_ can considerably dissolve in the solvent and, due to this; solvent also has a considerable effect on the interaction between CO_2_ and epoxide. By changing the solvent of the reaction, there was no considerable change in the conversion of epoxide to cyclic carbonate but, it was found that the rate of the reaction is higher in CH_3_CN which maybe is related to the better solubility of the reagents in this solvent. Higher dielectric constant of CH_3_CN (37.5) than other used solvents (THF = 7.58, CHCl_3_ = 4.81 and EtOH = 24.55) can be also influential in this process. The low boiling point of THF (66 °C) and CHCl_3_ (61.2 °C) in comparison to CH_3_CN (82 °C) and its limiting effect is also effective on the reaction rate in these solvents. It should be noted that 1-phenyl-1,2-ethanediol was obtained as a byproduct in ethanol solvent, which can be attributed to the epoxide ring opening reaction by the attack of the water molecules in this solvent. Therefore, due to the formation of byproduct and lower yield of the cyclic carbonate, ethanol was the worst solvent among the investigated ones.

**Table 4:** Chemical CO_2_ fixation by styrene epoxide in the presence of Si-[Zn(HL)(OAc)_2_] under different conditions^a^

Entry	TBAB (mmol)	Solvent	Temp. (°C)	Time (h)	Yield^b^ (%)	TON	TOF (h^−1^)
1	—	CH_3_CN	50	18	10	54	3.0
2	0.10	CH_3_CN	50	18	61	332	18.4
3	0.50	CH_3_CN	50	12	70	380	31.7
4	1.00	CH_3_CN	50	12	82	446	37.2
5	1.50	CH_3_CN	50	12	84	456	38.0
6	2.00	CH_3_CN	50	12	87	473	39.4
7	1.00	CH_3_CN	70	12	94	511	42.6
8	1.00	CH_3_CN	80	12	95	516	43.0
9	1.00	CH_3_CN	RT	12	46	250	20.8
10	1.00	THF	70	12	68	370	30.8
11	1.00	CHCl_3_	70	12	72	391	32.6
12	1.00	EtOH	70	12	66	359	29.9
13^c^	1.00	CH_3_CN	70	12	19	—	—

a Reaction condition: Si-[Zn(HL)(OAc)_2_] (15.0 mg, 9.20 μmol Zn(ii) ion), styrene epoxide (5.0 mmol), solvent (10 ml).

b Isolated yield of cyclic carbonate.

c Without catalyst.

When the catalytic reaction was finished, the product was extracted by solvent and Si-[Zn(HL)(OAc)_2_] was recovered from the reaction mixture by filtration. The remaining compound was characterized by FT-IR spectroscopy to investigate the stability of Si-[Zn(HL)(OAc)_2_] during the catalytic reaction. Considering the FT-IR spectrum of the recovered Si-[Zn(HL)(OAc)_2_] (Fig. S12†) indicated that it is comparable with the spectrum of the fresh one. This matter confirms that there is no considerable change in the structure and composition of Si-[Zn(HL)(OAc)_2_] during and after catalytic reaction and it has acceptable stability under the investigated catalytic reaction. The recovered catalyst showed acceptable activity in the next runs and it could efficiently catalyze the CO_2_ fixation reaction without meaningful decrease in its activity. Only 6% decrease in the conversion of substrate was observed after five recovering and reusing cycles. The catalytic activity of [Zn(HL)(OAc)_2_] with same loading of Zn(ii) ion (9.20 μmol, 4.0 mg) was also investigated in the optimum condition obtained for Si-[Zn(HL)(OAc)_2_] (Temp. = 70 °C, TBAB = 1.0 mmol, CH_3_CN = 10 ml). In this case the reaction was completed after about 8 hours which indicates [Zn(HL)(OAc)_2_] in homogeneous condition has higher catalytic activity than heterogenised Si-[Zn(HL)(OAc)_2_]. This matter is mainly related to higher interaction of catalyst with reagents in the homogeneous condition.

The catalytic activity of Si-[Zn(HL)(OAc)_2_] was compared with the activity of the other homogeneous and heterogeneous Zn(ii) coordination compounds (see Table 5) and the results indicated that this compound has moderate activity in comparison with the similar catalytic systems. The mechanism of the chemical CO_2_ fixation in the presence of Zn(ii) catalysts has been investigated in the literature.^34^ The proposed mechanism for chemical CO_2_ fixation reaction in the presence of Si-[Zn(HL)(OAc)_2_] is illustrated in Scheme S1.† By considering the structure and composition of Si-[Zn(HL)(OAc)_2_] and also the results of the catalytic reactions, it is predictable that the mechanism of this system is similar to the previous reports and the cyclic carbonate produces by the cyclization reaction of CO_2_ with activated epoxide by TBAB co-catalyst in the presence of Zn(ii) ion.

**Table 5:** Comparing catalytic activity of Si-[Zn(HL)(OAc)_2_] with some of the similar catalytic systems based on Zn(ii) compounds

Catalyst	Co-catalyst	Pressure	Temp. (°C)	Time (h)	Yield (%)	TOF (h^−1^)	Ref.
Si-[Zn(HL)(OAc)_2_]	TBAB	1 atm	70	12	94	42.6	This study
La–Zn complex with diglycolamine-bis(phenolate) ligand	TBAB	1 atm	25	24	93	7.8	35
Zn porphyrin complex	TBAB	1 atm	70	4	99>	13.88	36
ZnL_2_ (*L* = 4,5-diphenyl-1*H*-imidazole-2-yl)phenol derivatives)	TBAI	2 MPa	110	5	93.1	186.2	37
[Zn(Me_6_Tren)ClO_4_]ClO_4_ (Me_6_Tren = tris[2-(dimethylamino)ethyl]amine	TBAI	1 bar	80	6	85	14.2	38
[Zn(Me_6_Tren)NO_3_]NO_3_	TBAI	1 bar	80	6	77	12.8	38
ZnTCPP (TCPP = 5,10,15,20-tetrakis(4-carboxyphenyl)-porphyrinato)	TBAB	1 bar	40	14	52.8	3.97	39
Zn(OH-salC_2_NH_2_Am) (OH-salC_2_NH_2_Am = *N*,*N*-diethyl-2-((2,3,4-trihydroxybenzylidene)amino)ethanaminium bromide)	TBAB	0.1 MPa	120	12	90	15	40
[PS-Zn(ii)L] (polystyrene supported Zn(ii) complex with bis-phenolate ligand)	TBAB	0.1 MPa	RT	8	98	21.9	41
ZnCl_2_	TBAI	1 bar	RT	24	27	11.3	42
ZnL^APIP^OAc (L^APIP^ = 2,4-di-*tert*-butyl-6-(((*E*)-2-(((*E*)-pyridin-2-lmethylene)amino) benzylidene)-amino)phenol)	TBAB	0.1 MPa	70	4	40	100	43
ZnMOF-1	TBAB	1 atm	40	8	68	4.7	44
{[(CH_3_)_2_NH_2_][Zn_2_(L)(H_2_O)PO_4_]·2DMF}_*n*_ (H_2_L = 5-(1*H*-imidazole-1-yl)isophthalic acid)	TBAB	1 atm	70	12	99	4.5	45

## 4. Conclusion

In summary, a new Zn(ii) coordination compound containing a free amine functionality on the ligand, [Zn(HL)(OAc)_2_] (1), was synthesized and characterized by several methods. The Zn(ii) ion in this compound is five coordinated and its coordination environment is created by coordination of ONN-donor atom of organic ligand and two oxygen atoms of acetate anions. The compound was successfully supported on the functionalized silica gel by using amidification reaction and the resulting heterogeneous catalyst, Si-[Zn(HL)(OAc)_2_], was characterized by atomic absorption, TGA, FT-IR, XRD, SEM, EDX and DRS analyses. The results confirmed that compound 1 is successfully supported on the silica, and it has high stability during and after catalytic reactions. The catalytic activity of Si-[Zn(HL)(OAc)_2_] was investigated in chemical CO_2_ fixation reaction and production of cyclic carbonate by using styrene epoxide as a model substrate. Si-[Zn(HL)(OAc)_2_] showed catalytic properties in the formation of cyclic carbonate and the results indicated that temperature, solvent and co-catalyst can improve the catalytic activity of this compound.

## Data availability

The data supporting this article have been included as part of the ESI.† Crystallographic data for the reported compound have been deposited at the Cambridge Crystallographic Data Center (CCDC No. 2409956) and can be obtained free of charge from https://www.ccdc.cam.ac.uk/structures/ and also from the online version of this article at https://doi.org/10.1039/D4RA09026H.

## Conflicts of interest

There are no conflicts to declare.

## Supplementary Material

RA-015-D4RA09026H-s001

RA-015-D4RA09026H-s002
