# Supplementary material for: Chemical CO2 fixation by a heterogenised Zn(ii)-hydrazone complex
Source: RSC Adv. 2025 Feb 26;15(8):5977–88. doi: 10.1039/d4ra09026h (PMC11862881; doi:10.1039/d4ra09026h)
Supplement: RA-015-D4RA09026H-s001 [file RA-015-D4RA09026H-s001.pdf]

Supplementary Information (SI) for RSC Advances.
This journal is © The Royal Society of Chemistry 2025

Supporting information file

Chemical CO_2 fixation by a heterogenised Zn(II)-hydrazone complex

Neda Heydari,[a] Rahman Bikas,[a,*] Tadeusz Lis[b]

[a] *Department of Chemistry, Faculty of Science, Imam Khomeini International University, 34148-96818, Qazvin, Iran*

[b] *Faculty of Chemistry, University of Wroclaw, Joliot-Curie 14, Wroclaw 50-383, Poland*

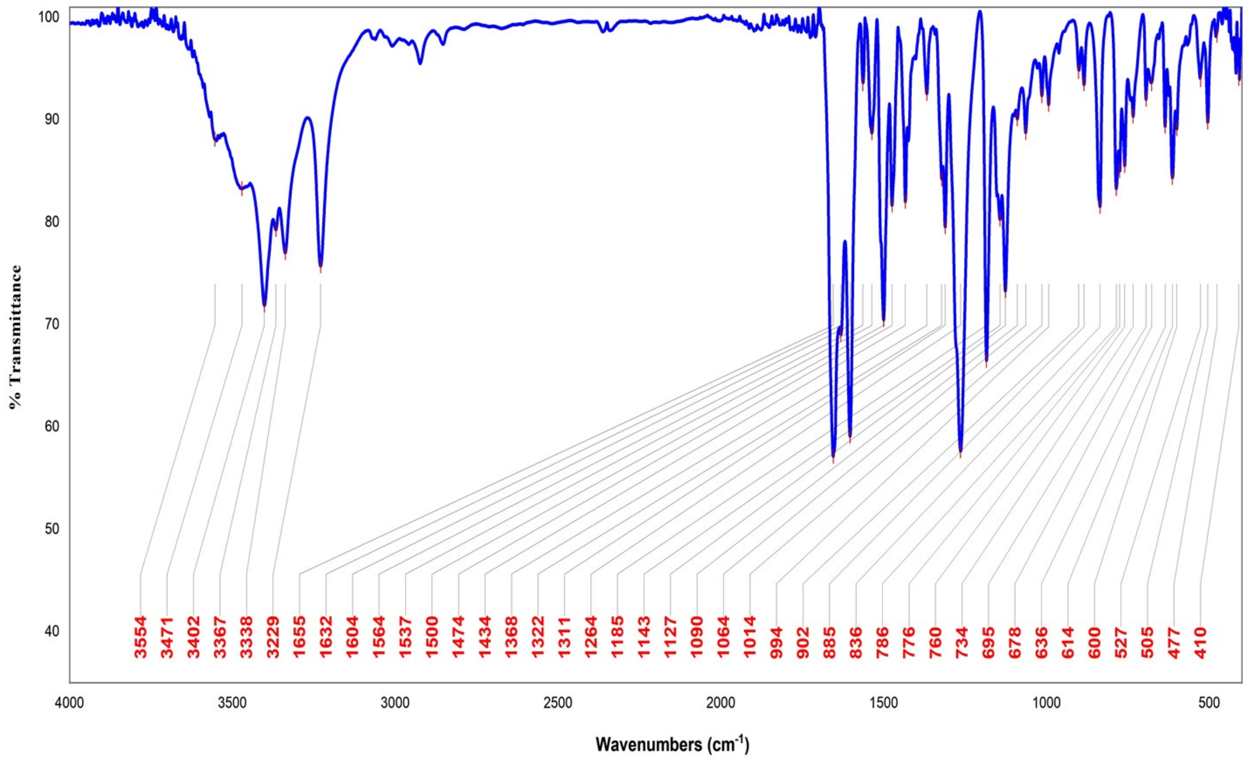

Fig. S1. FT-IR spectrum of HL

* Email: bikas@sci.ikiu.ac.ir; bikas_r@yahoo.com

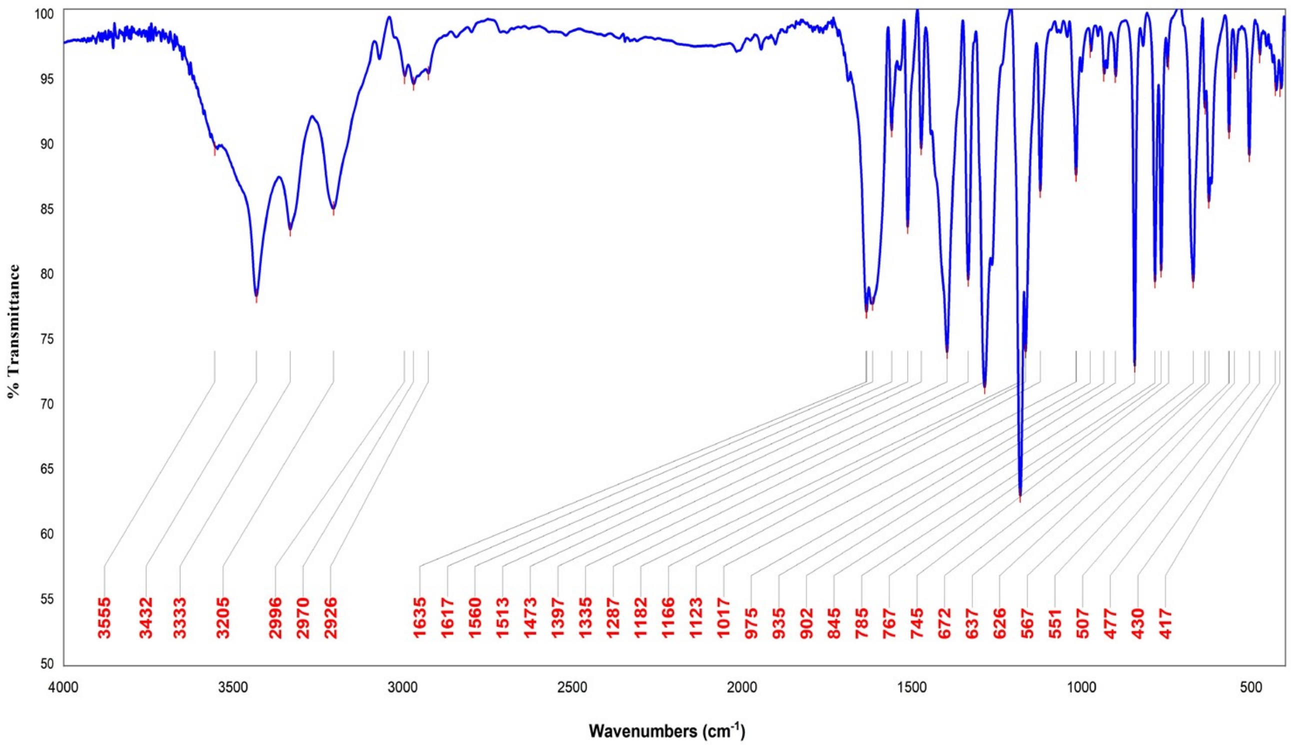

Fig. S2. FT-IR spectrum of $[Zn(HL)(OAc)_2]$

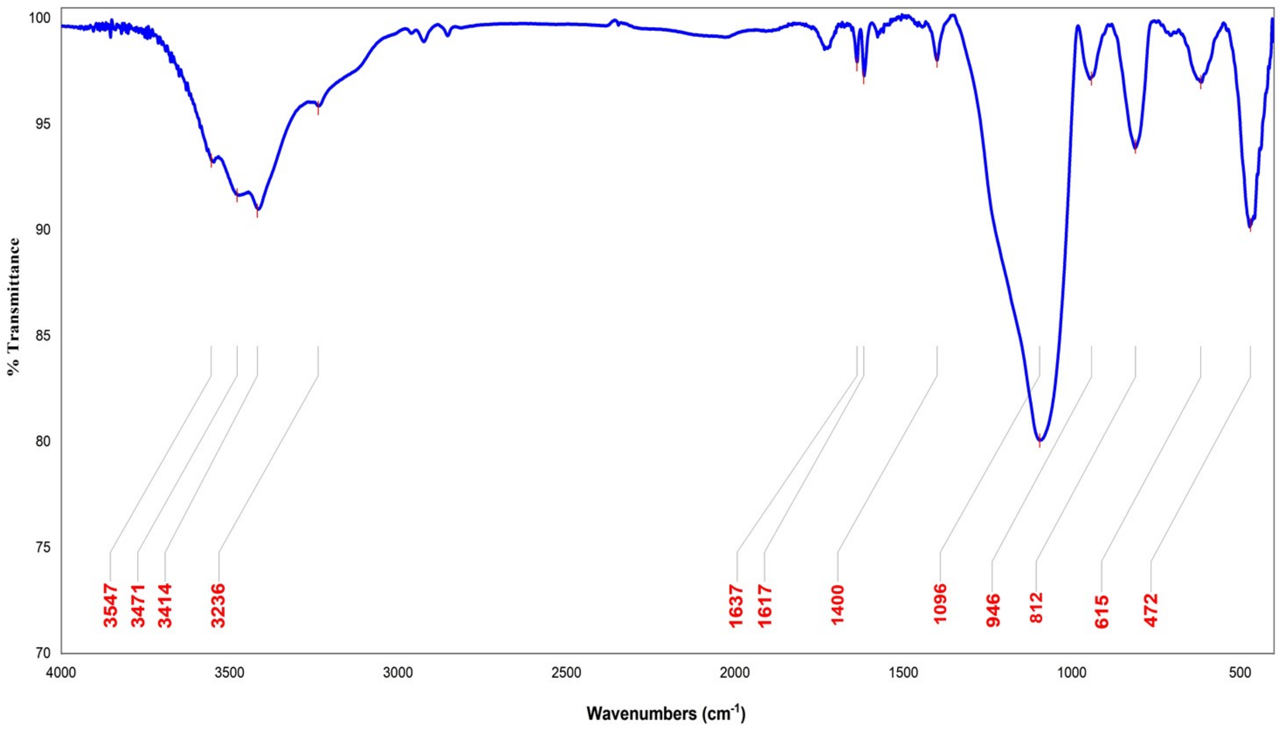

Fig. S3. FT-IR spectrum of propionylchloride-functionalized silica gel

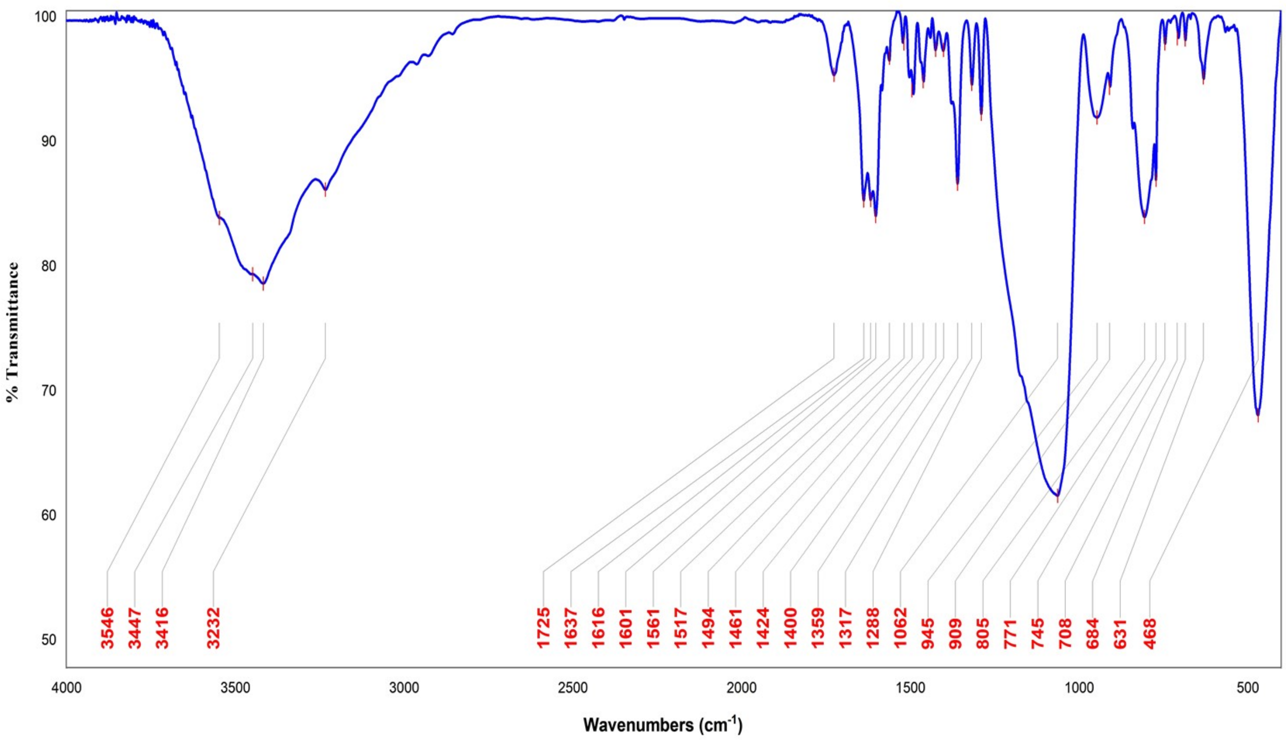

Fig. S4. FT-IR spectrum of **Si-[Zn(HL)(OAc)$_2$]**

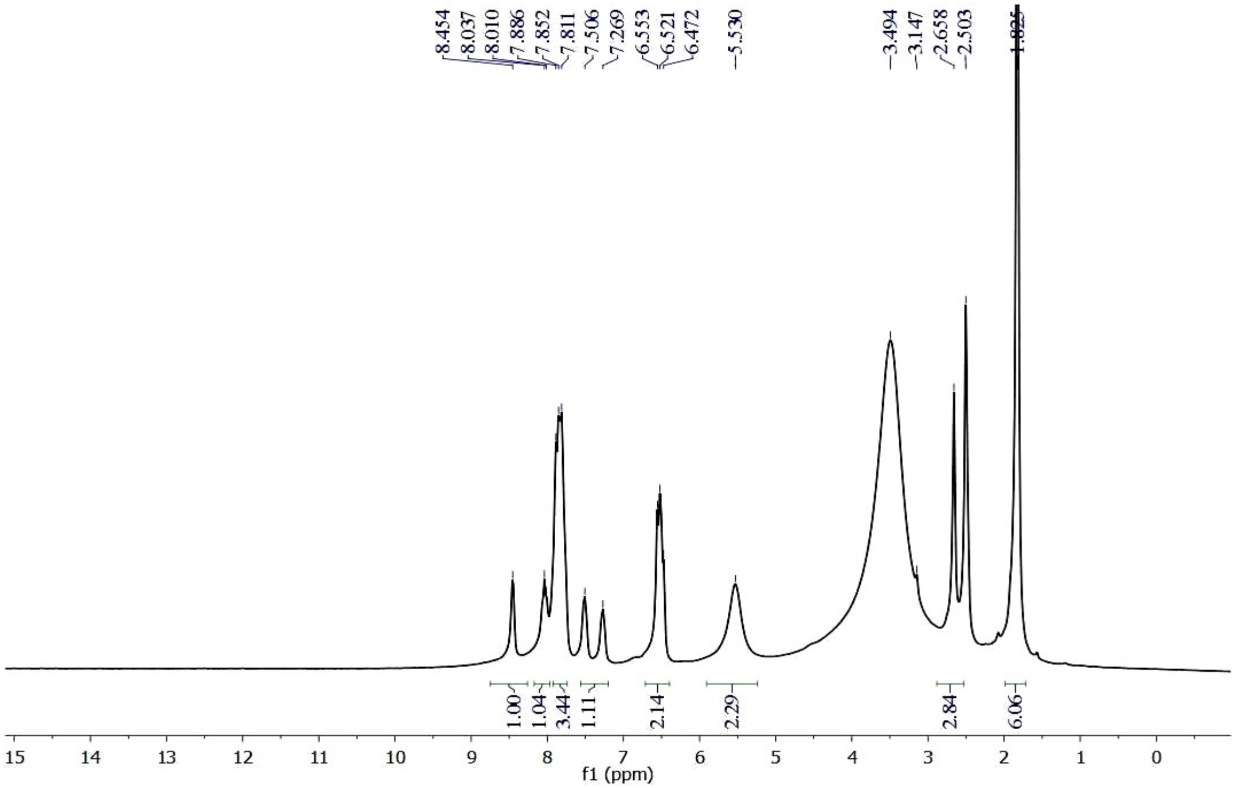

Fig. S5. ^{1}H NMR spectrum of [Zn(HL)(OAc)$_2$] in DMSO-d$_6$

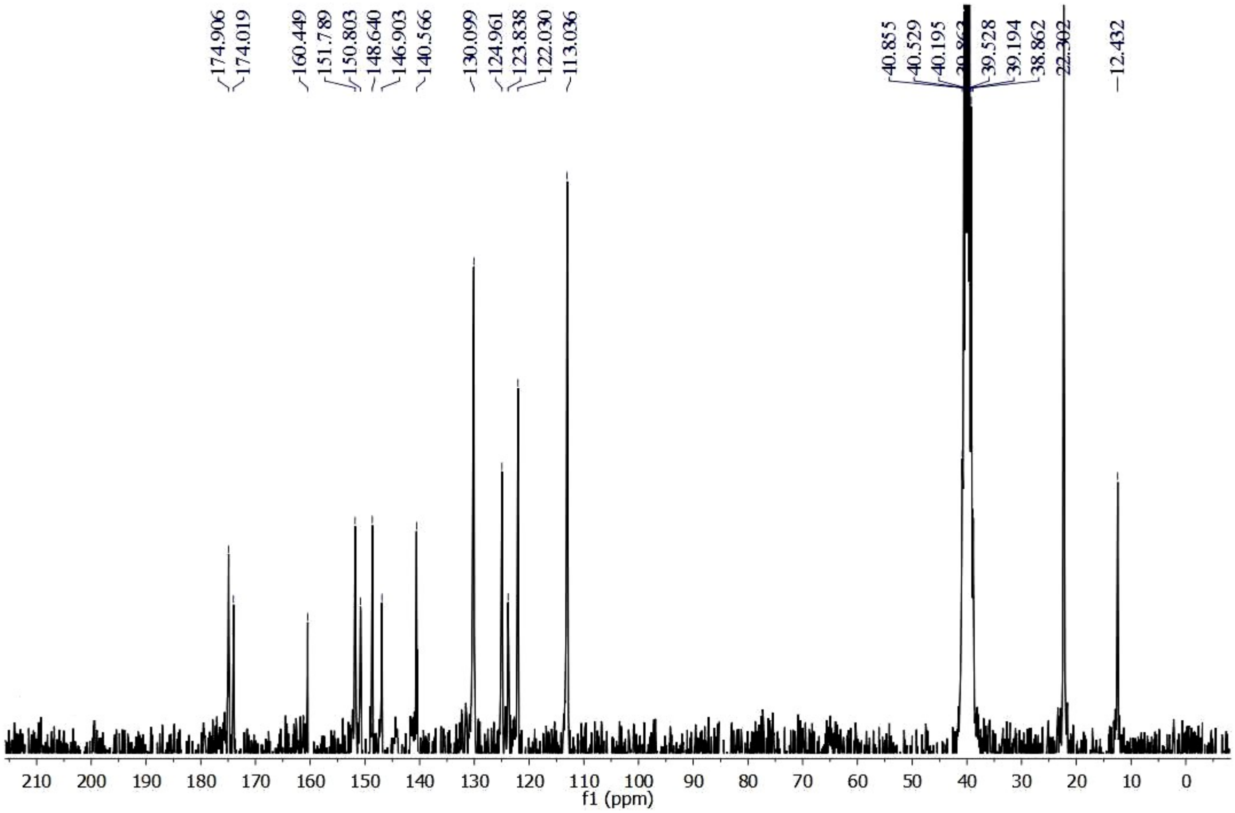

Fig. S6. ^{13}C NMR spectrum of $[Zn(HL)(OAc)_2]$ in DMSO-d_6

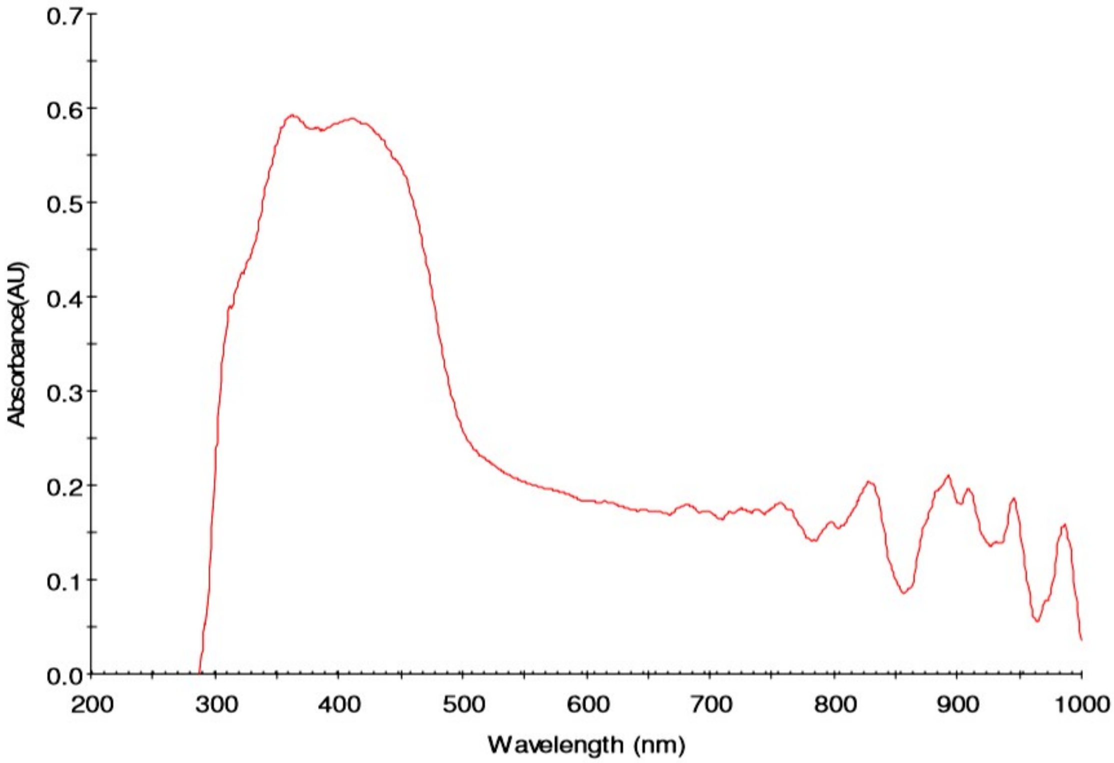

Fig. S7. UV-DRS spectrum of **Si-$[Zn(HL)(OAc)_2]$**

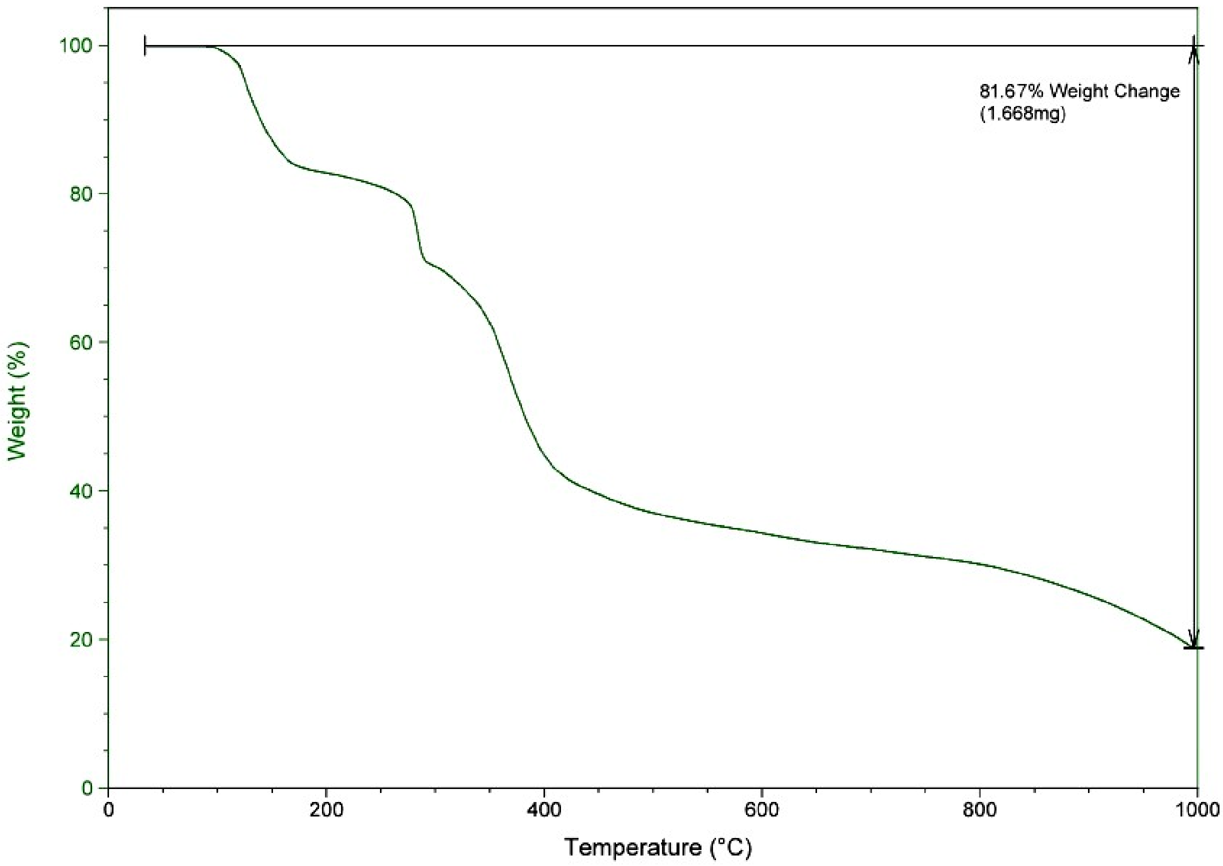

Fig. S8. TGA diagram of $[Zn(HL)(OAc)_2]$

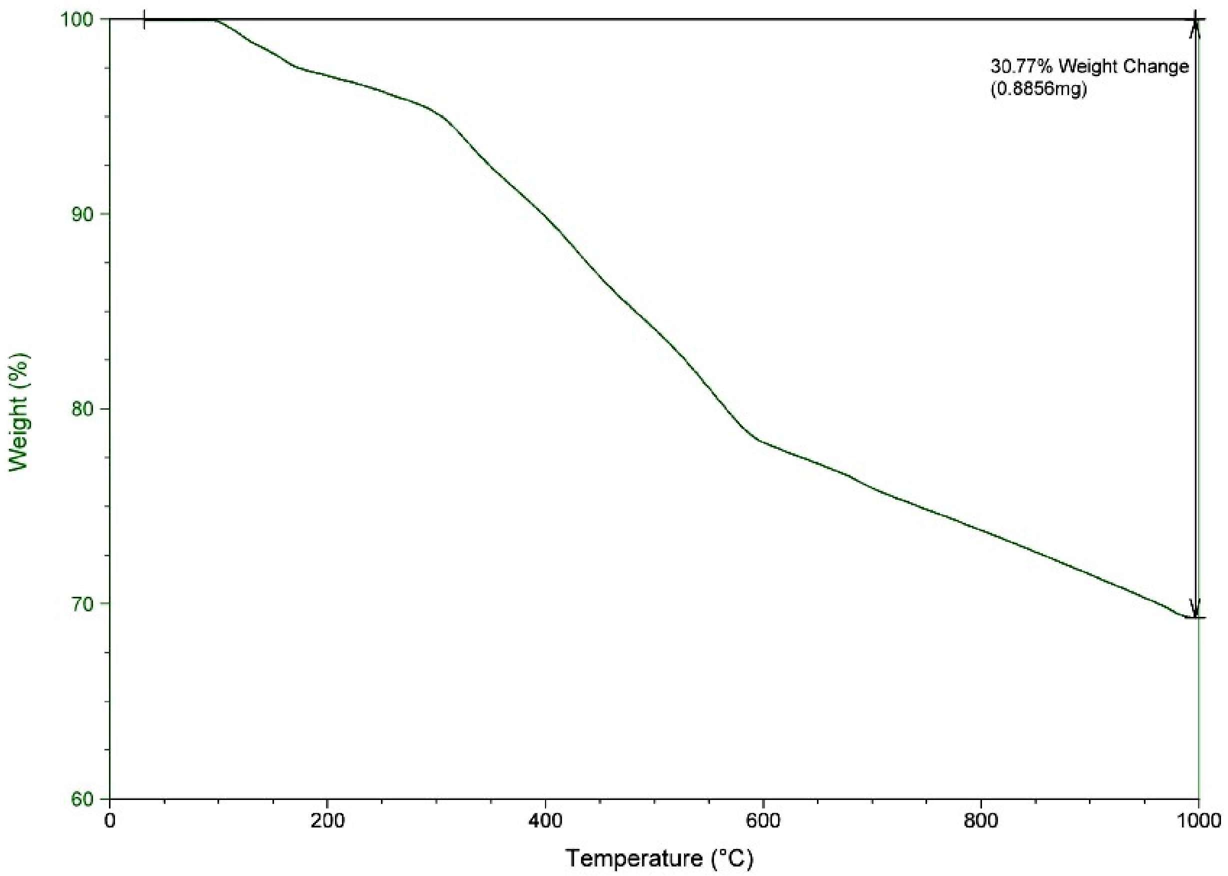

Fig. S9. TGA diagram of **Si-[Zn(HL)(OAc)$_2$]**

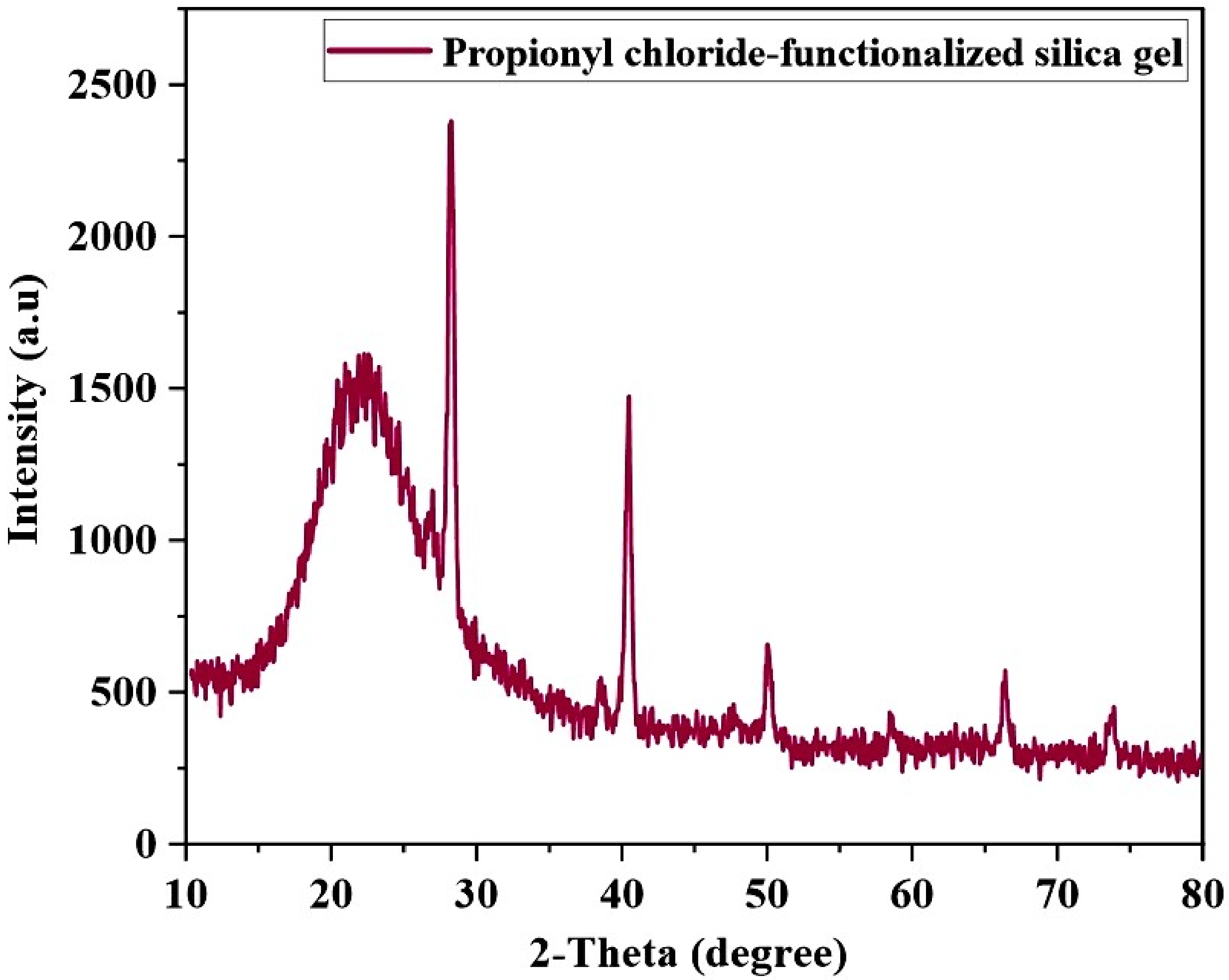

Fig. S10. XRD pattern of propionylchloride-functionalized silica gel

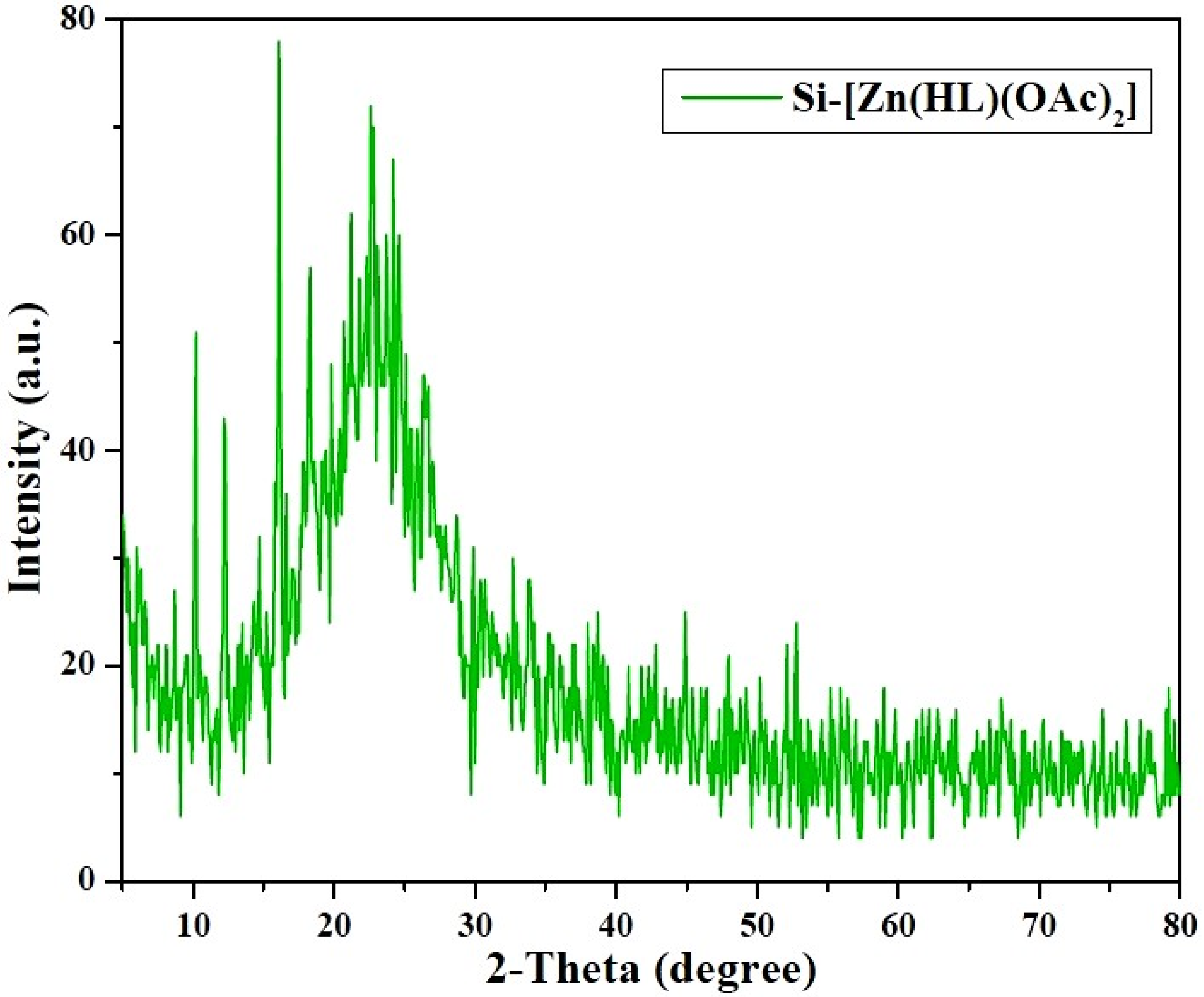

Fig. S11. XRD pattern of **Si-[Zn(HL)(OAc)$_2$]**

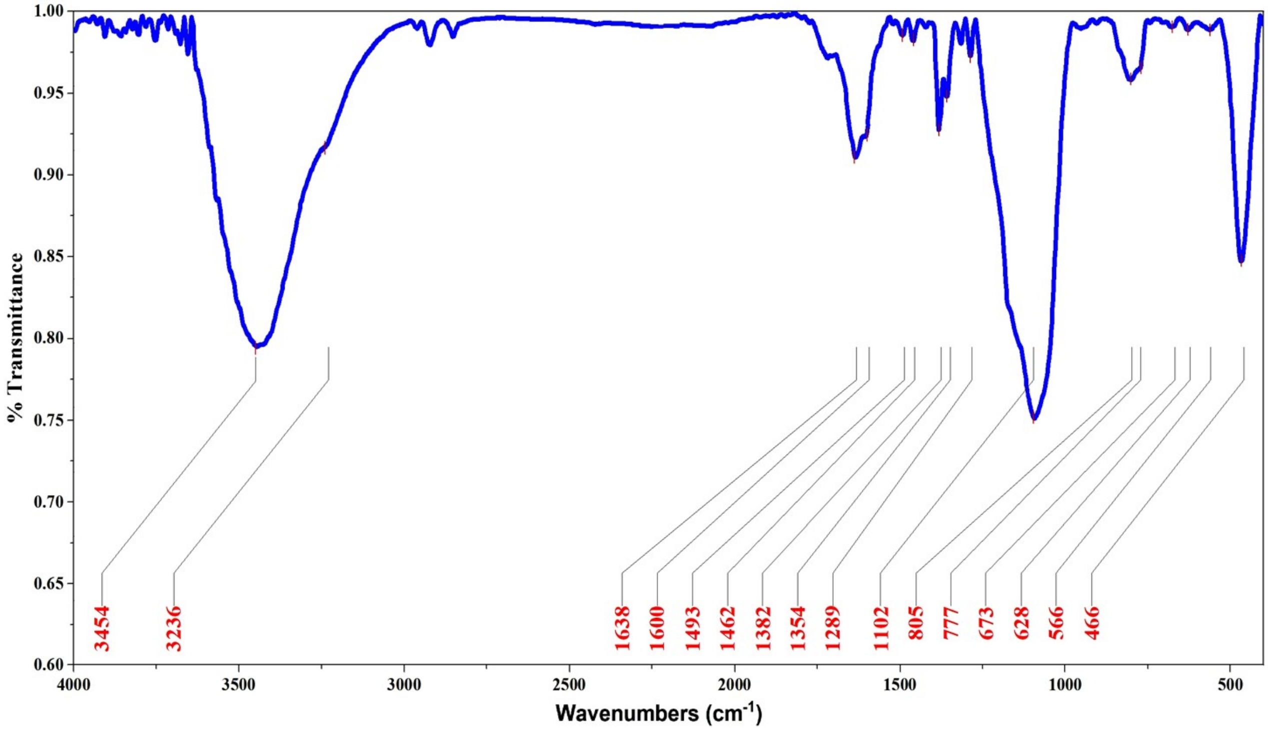

Fig. S12. FT-IR spectrum of the recovered **Si-[Zn(HL)(OAc)$_2$]**

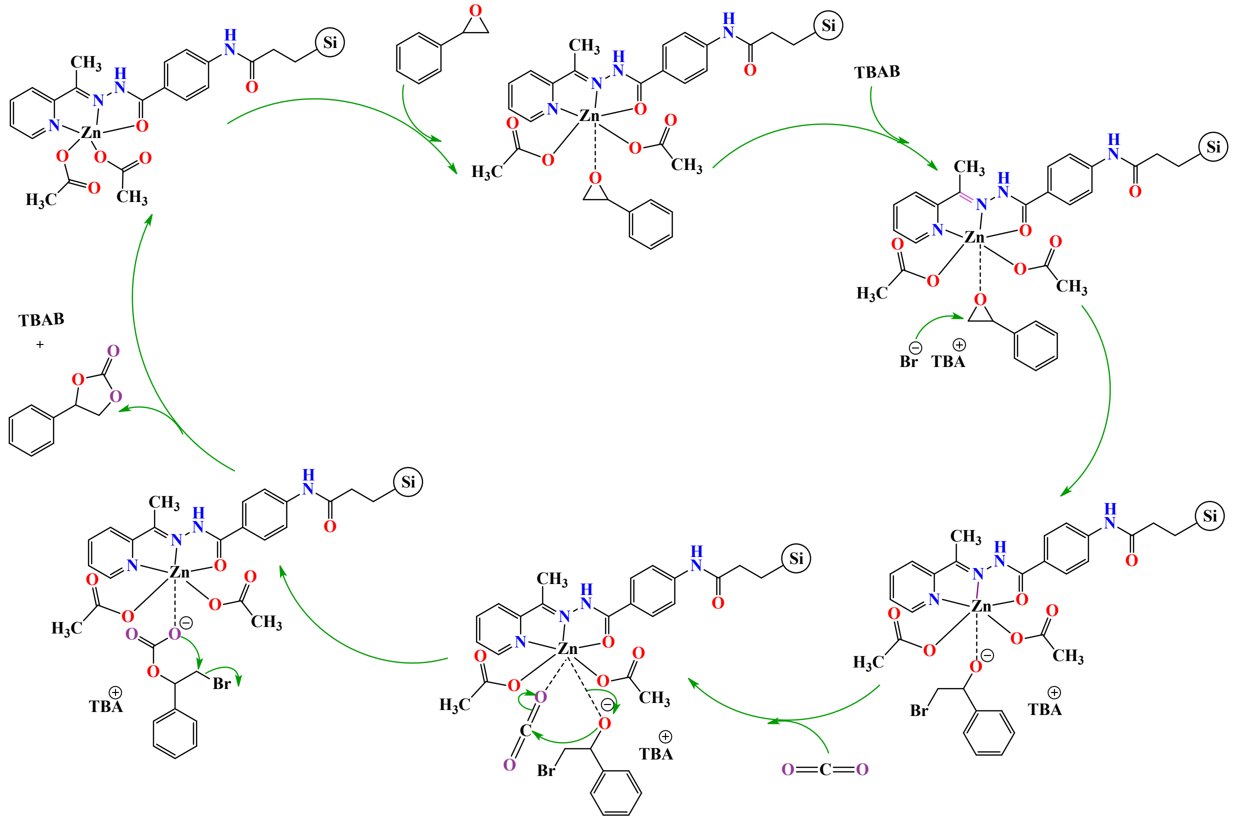

Scheme S1. The proposed mechanism for chemical CO_2 fixation reaction in the presence of **Si-[Zn(HL)(OAc)$_2$]**
